# Repurposing Cofilin-Targeting Compounds for Ischemic Stroke Through Cheminformatics and Network Pharmacology

**DOI:** 10.3390/ph18091323

**Published:** 2025-09-04

**Authors:** Saleh I. Alaqel, Abida Khan, Mashael N. Alanazi, Naira Nayeem, Hayet Ben Khaled, Mohd Imran

**Affiliations:** 1 Department of Pharmaceutical Chemistry, College of Pharmacy, Northern Border University, Rafha 91911, Saudi Arabia; 2 King Salman Center for Disability Research, Riyadh 11614, Saudi Arabia; 3 Center for Health Research, Northern Border University, Arar 73213, Saudi Arabia

**Keywords:** cofilin, stroke, *LIMK1*, drug repurposing, QSAR, network pharmacology

## Abstract

**Background/Objectives**: Cofilin, a key regulator of actin cytoskeleton dynamics, contributes to neuroinflammation, synaptic damage, and blood–brain barrier disruption in ischemic stroke. Despite its established role in stroke pathology, cofilin remains largely untargeted by existing therapeutics. This study aimed to identify potential cofilin-binding molecules by repurposing *LIMK1* inhibitors through an integrated computational strategy. **Methods**: A cheminformatics pipeline combined QSAR modeling with four molecular fingerprint sets and multiple machine learning algorithms. The best-performing QSAR model (substructure–Random Forest) achieved R^2^_train = 0.8747 and R^2^_test = 0.8078, supporting the reliability of compound prioritization. Feature importance was assessed through SHAP analysis. Top candidates were subjected to molecular docking against cofilin, followed by 300 ns molecular dynamics simulations, MM-GBSA binding energy calculations, principal component analysis (PCA), and dynamic cross-correlation matrix (DCCM) analyses. Network pharmacology identified overlapping targets between selected compounds and stroke-related genes. **Results**: Three compounds, CHEMBL3613624, ZINC000653853876, and Gandotinib, were prioritized based on QSAR performance, binding affinity (−6.68, −6.25, and −5.61 Kcal/mol, respectively), and structural relevance. Docking studies confirmed key interactions with Asp98 and His133 on cofilin. Molecular dynamics simulations supported the stability of these interactions, with Gandotinib showing the highest conformational stability, and ZINC000653853876 exhibiting the most favorable energetic profile. Network pharmacology analysis revealed eight intersecting targets, including *MAPK1*, *PRKCB*, *HDAC1*, and serotonin receptors, associated with neuroinflammatory and vascular pathways in strokes. **Conclusions**: This study presents a rational, integrative repurposing framework for identifying cofilin-targeting compounds with potential therapeutic relevance in ischemic stroke. The selected candidates warrant further experimental validation.

## 1. Introduction

Stroke remains a major cause of long-term disability and mortality worldwide, with ischemic stroke accounting for most cases. A defining feature of neuronal injury following ischemia is the disruption of the actin cytoskeleton [1,2]. This breakdown contributes to blood–brain barrier dysfunction, neuronal cell death, and impaired synaptic connectivity [3]. While actin and tubulin are essential for maintaining cytoskeletal stability, they primarily act as static scaffolds and are not rapidly regulated in response to ischemic stress. In contrast, cofilin functions as a dynamic regulator of actin turnover, becoming quickly activated during ischemic conditions and directly triggering cytoskeletal collapse, mitochondrial dysfunction, and neuronal apoptosis. This uniquely regulated role makes cofilin a more immediate and modifiable target for therapeutic intervention in stroke pathogenesis. Cofilin-1, a key regulator of actin dynamics, belongs to the actin-depolymerizing factor (ADF)/cofilin family and plays a central role in filament severing and turnover [4]. Under pathological conditions, aberrant activation and accumulation of cofilin have been linked to actin rod formation, mitochondrial dysfunction, and neurodegeneration, particularly during ischemic stress [5,6]. These pathological aggregates impair actin turnover and interfere with intracellular trafficking and energy metabolism.

Cofilin’s activity is tightly regulated by phosphorylation at the Ser3 residue, mediated by LIM kinase 1 (*LIMK1*). In its phosphorylated form, cofilin is inactive and cannot bind actin. Upon dephosphorylation, it regains actin-binding capacity, which under ischemic conditions contributes to cytoskeletal destabilization [7,8,9]. This upstream regulation has made *LIMK1* a pharmacological target in several neurological disorders. However, efforts to modulate cofilin indirectly through *LIMK1* inhibition have shown limited therapeutic benefit, partly due to feedback regulation, off-target effects, and poor brain permeability of existing *LIMK1* inhibitors. While multiple *LIMK1* inhibitors have been developed, their ability to bind cofilin directly has not been systematically explored. Drug repurposing offers a strategy to identify new targets for known compounds, especially those originally designed for cytoskeletal modulation. *LIMK1* inhibitors contain functional groups capable of engaging polar and charged residues, features that are common on the cofilin surface [9,10,11,12,13]. Given the lack of known scaffolds specifically designed to target cofilin, this study aimed to evaluate *LIMK1* inhibitors as repurposing candidates through a focused computational pipeline.

An integrated in silico framework was used to identify *LIMK1* inhibitors that may bind directly to cofilin. The study combined ligand-based QSAR modeling with molecular docking, molecular dynamics simulations, and network pharmacology analysis. Machine learning models were trained using various descriptor sets to define structure–activity relationships in a curated dataset of *LIMK1* inhibitors. Feature attribution methods were applied to prioritize hits based on chemical patterns relevant to binding. Docking simulations were used to assess the compatibility of selected compounds with cofilin’s active groove, focusing on residues known to mediate actin binding. To evaluate binding persistence and structural adaptability, molecular dynamics simulations were conducted on selected complexes. These were further analyzed through MM-GBSA binding energy calculations, principal component analysis (PCA), and dynamic cross-correlation matrices (DCCM) to examine energetics and motion. A network pharmacology approach was then applied to map compound-associated targets to stroke-related genes, identifying shared pathways and clusters with functional relevance. The aim of this work is to assess whether *LIMK1* inhibitors can be redirected toward direct cofilin modulation and to evaluate their potential relevance to ischemic stroke. By combining cheminformatics, structure-based modeling, and systems-level mapping, this study offers a rational strategy to advance cofilin-targeting agents and provides a foundation for future translational exploration in stroke therapy.

## 2. Results

### 2.1. QSAR Model Performance and Statistical Validation

To develop predictive models targeting *LIMK1* inhibitors with potential repurposing activity against cofilin signaling in stroke, a dataset of 204 molecules was compiled and filtered based on their bioactivity values. Molecules with pIC_50_ ≥ 7 were labeled as active and those with pIC_50_ < 6 as inactive. Intermediate compounds were excluded from modeling. After this stratification, 166 molecules were used for training and 38 for testing. The training set comprised 63 actives (38%) and 103 inactives (62%), while the test set included 19 actives (50%) and 19 inactives (50%), ensuring balanced class representation for model evaluation. The independent test set was reserved solely for final performance evaluation, with all cross-validation and hyperparameter tuning performed exclusively on the training set. Molecular descriptors were generated using PaDEL, and four distinct sets, MACCS (74), PubChem (187), CDK (819), and substructure (16), were retained after low-variance filtering. Each descriptor set was used to train eight machine learning algorithms: Random Forest, Support Vector Regression (SVR), Gradient Boosting, K-Nearest Neighbors (KNNs), Bagging, Ridge Regression, Partial Least Squares (PLS), and Gaussian Process Regression. Model performance was assessed based on regression statistics (R, R^2^, RMSE, MAE) and binary classification metrics (precision, recall, specificity, F1 score), ensuring interpretability and generalization across models (Table 1). MACCS descriptors paired with Bagging produced strong and balanced predictive performance, achieving perfect precision (1.000) with an F1 score of 0.812 and recall of 0.684. This model also achieved a test R^2^ of 0.764 with an RMSE of 0.673, indicating consistent regression accuracy. These results suggest that Bagging can effectively capture the non-linear relationships in the MACCS fingerprint space while avoiding overfitting.

**Figure 1:**
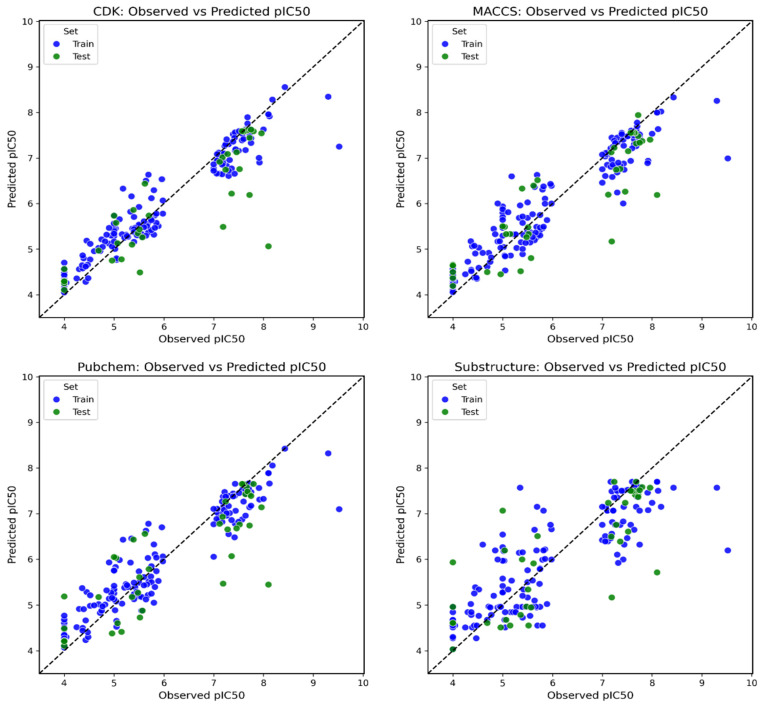
Observed vs. predicted pIC_50_ plots for CDK, MACCS, PubChem, and substructure descriptor-based QSAR models showing good agreement across training and test sets.

CDK descriptors combined with Ridge Regression demonstrated stable regression and classification performance, with a test R^2^ of 0.674, RMSE of 0.792, and an F1 score of 0.882 (recall = 0.789, precision = 1.000). The inherent regularization in Ridge Regression likely mitigated the high dimensionality of CDK features, leading to robust generalization on the test set. PubChem descriptors with Gradient Boosting also yielded competitive results, achieving an F1 score of 0.774 (precision = 1.000, recall = 0.632) and a test R^2^ of 0.661 with an RMSE of 0.807. While recall was lower compared to the MACCS–Bagging and CDK–Ridge models, the high precision suggests that the model reliably identified actives, though some may have been missed. Substructure fingerprints combined with Gradient Boosting achieved an F1 score of 0.882, with a recall and precision of 0.789 and 1.000, respectively. Despite having only 16 descriptors, the model performed competitively in both regression (test R^2^ = 0.662, RMSE = 0.806) and classification metrics, indicating that these compact descriptors effectively captured essential structure–activity relationships. Across all tested combinations, Gaussian Process models consistently showed instability between training and testing, suggesting overfitting, especially in descriptor-rich datasets. Considering both regression and classification perspectives, the four models, MACCS–Bagging, CDK–Ridge Regression, PubChem–Gradient Boosting, and substructure–Gradient Boosting, demonstrated the most reliable and balanced performance (Figure 1). The complete metrics for all tested models are available in the Supplementary Materials (Tables S1–S13).

### 2.2. Cross-Validation and Selection of Hit Models

Using the training set described in Section 2.1, we performed 10-fold cross-validation to assess model generalization and guide hyperparameter tuning, ensuring the test set remained untouched for unbiased evaluation. This baseline assessment revealed that PubChem and CDK descriptors consistently supported models with better predictive stability, particularly in Random Forest and SVR algorithms (Table 2 and Table S14). For instance, the PubChem–Random Forest model achieved an average R^2^ of 0.5768 ± 0.2296, while the CDK–SVR model reported a similar performance at 0.5805 ± 0.2233. These R^2^ values, paired with relatively lower RMSE and MAE, indicated a strong model fit and low prediction error even before optimization. Substructure-based models demonstrated moderate performance, with SVR and Gradient Boosting yielding R^2^ values of 0.5276 ± 0.2665 and 0.4772 ± 0.3235, respectively. Despite the smaller descriptor set (n = 16), their performance was competitive and highlighted the utility of well-defined structural fingerprints in learning relevant activity trends without overfitting. MACCS-based models, while slightly lower in pre-tuning performance, offered acceptable regression metrics and strong potential for improvement under optimized settings. In contrast, some KNN-based models exhibited higher variability, particularly on MACCS and CDK, suggesting sensitivity to local patterns or outliers.

Following this initial evaluation, hyperparameter tuning was conducted using grid search with 5-fold cross-validation, and the best parameter sets were then subjected to a second round of 10-fold validation. Notable improvements were observed across most models. The PubChem–Random Forest model, for example, improved its R^2^ mean to 0.5826 ± 0.2291 with reduced RMSE (0.8107) and MAE (0.6184), reinforcing its robustness as a high-dimensional descriptor set. CDK–SVR maintained its strong performance (R^2^ = 0.5805 ± 0.2233), reflecting the algorithm’s adaptability in managing descriptor complexity. The MACCS–Random Forest model also demonstrated enhanced predictive stability post-tuning (R^2^ = 0.5575 ± 0.2120), and the substructure–SVR model reached an R^2^ of 0.5285 ± 0.2487. These results confirmed that both low-dimensional and moderate-dimensional fingerprint sets could yield dependable predictions when paired with appropriately tuned models. To ensure the interpretability and reliability of subsequent predictions, we selected six top-performing models based on the overall consistency between training metrics, validation performance, and cross-validation robustness. These included Random Forest and SVR for substructure, Random Forest for MACCS and PubChem, and both Random Forest and SVR for CDK. These selected models showed a combination of high R^2^, minimized error margins, and acceptable standard deviations, thereby making them suitable for subsequent domain applicability and mechanistic interpretation studies.

### 2.3. Applicability Domain Assessment Using William’s Plot

**Figure 2:**
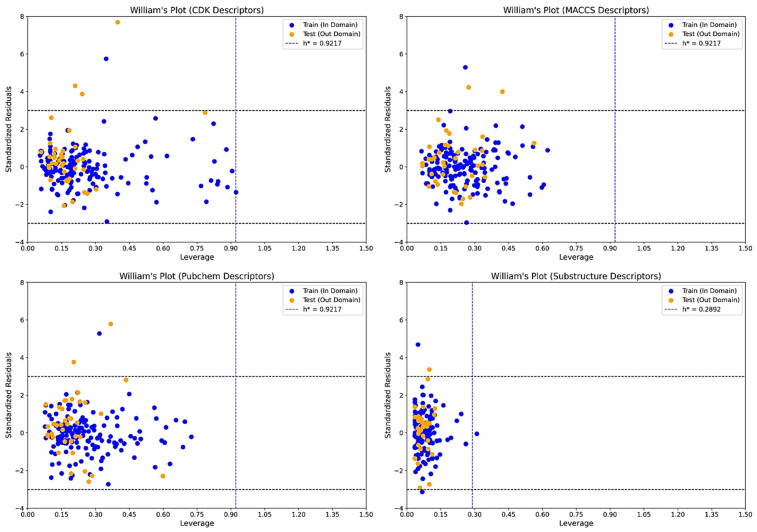
William’s plots for CDK, MACCS, PubChem, and substructure descriptors showing the applicability domain of QSAR models. The blue vertical line (h*) indicates the leverage threshold, and the black horizontal lines (±3) denote residual limits. Most compounds fall within these boundaries, supporting model reliability. Orange and blue dots represent test and training set compounds, respectively, with points outside the boundaries considered outliers.

**Figure 3:**
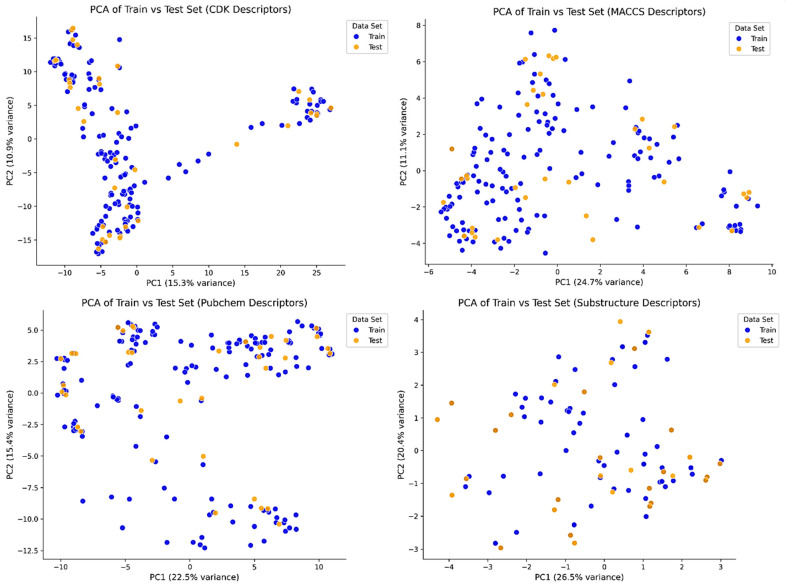
PCA plots of train and test sets using CDK, MACCS, PubChem, and substructure descriptors. Blue and orange dots represent train and test compounds, respectively. Overlap across PC1 and PC2 indicates similar chemical space distribution, supporting model reliability.

To assess the generalization ability and prediction confidence of the developed QSAR models, William’s plot analysis was performed for each descriptor set using their respective top-performing algorithms. These plots visualize standardized residuals against leverage, allowing the identification of both statistically influential compounds (high leverage) and activity outliers (residuals > ±3). The leverage threshold (h*) was calculated for each model, and compounds exceeding this threshold or the residual boundary were interpreted as potential outliers (Figure 2). PCA was performed on each descriptor set to visualize the distribution of training and test compounds in reduced dimensional space and assess dataset representativeness. For CDK descriptors, the data showed high structural diversity with well-mixed clustering, supporting generalization but with a broader chemical space. MACCS and substructure descriptors displayed tighter and more centralized clustering with good overlap between training and test sets, suggesting consistent structural representation and lower variance. PubChem descriptors showed wide dispersion, indicating descriptor richness but possible inclusion of noisy or redundant features (Figure 3).

The substructure–Random Forest model showed a tightly bound applicability domain with an h* value of 0.2892. Most compounds, including both training and test sets, fell well within the boundary, indicating consistent predictability. Only three compounds, CHEMBL3410034 (train), CHEMBL5278261 (train), and CHEMBL5275515 (test), were identified as outliers. CHEMBL3410034 and CHEMBL5278261 had standardized residuals beyond ±3, and the latter appeared across multiple descriptor sets, confirming it as structurally and statistically unique. The model’s conservative domain is likely attributed to the lower complexity of the substructure fingerprint set (16 descriptors), reducing variance and overfitting. The CDK–Random Forest model, based on 819 descriptors, presented a broader domain (h* = 0.9217), capturing a wider chemical space. Three outlier compounds were detected in the test set CHEMBL5206269, CHEMBL3410055, and CHEMBL5275515 with standardized residuals as high as 7.68 in the latter. One training set compound, CHEMBL5278261, was also flagged. These molecules exhibited extreme predicted deviations, indicating that despite the model’s general stability, CDK-based predictions should be interpreted cautiously for molecules lying at structural extremes.

The MACCS–Random Forest model (74 descriptors; h* = 1.3554) also showed moderate boundary adherence. Three outliers emerged in the training set: CHEMBL5278261, CHEMBL5206269, and CHEMBL5275515. Although most predictions were contained within the acceptable range, these repeated outliers highlight specific chemotypes not adequately captured by MACCS features. Nonetheless, the remaining dataset showed minimal dispersion, indicating a good balance between generalization and structural coverage. The PubChem–Random Forest model, composed of 187 descriptors, exhibited the broadest applicability domain among the four sets. The calculated h* value was 0.9217, matching that of CDK. Outliers were found in both training and test sets. Specifically, CHEMBL5278261 (train), CHEMBL5206269 (test), and CHEMBL5275515 (test) displayed extreme deviations, again suggesting overprediction for these structural variants. The wider chemical space encompassed by PubChem descriptors may have introduced noise or redundancy, contributing to these anomalies despite model tuning. Across all descriptor sets, three compounds, CHEMBL5278261, CHEMBL5275515, and CHEMBL5206269, recurred as outliers, making them chemically and statistically divergent from the core dataset. CHEMBL5278261 was consistently flagged in every model’s training set, while CHEMBL5275515 and CHEMBL5206269 appeared as test set outliers in at least two models. These compounds likely represent scaffold or property extremes not captured well by the molecular descriptors used, warranting caution during interpretation or experimental follow-up. Thus, William’s plot analysis provided crucial insights into model reliability and chemical coverage. While substructure and MACCS-based models showed tighter clustering and fewer leverage violations, models trained on CDK and PubChem descriptors covered broader chemical spaces with greater boundary uncertainty. These evaluations reinforce the importance of integrating applicability domain checks before progressing to prediction-based molecular screening and decision-making. The full report is compiled in the Supplementary Materials (Tables S15–S22).

### 2.4. Feature Importance and Interpretability Using SHAP Analysis

**Figure 4:**
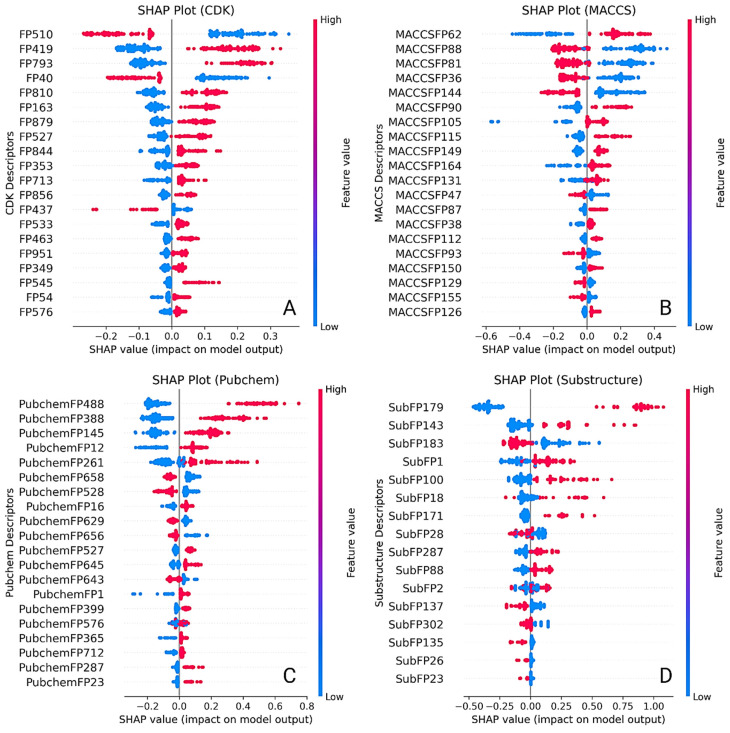
SHAP value analysis illustrating feature importance for QSAR models built using different descriptor sets: (**A**) CDK, (**B**) MACCS, (**C**) PubChem, and (**D**) substructure. Each plot highlights the top contributing features driving predicted pIC_50_ values. Points are colored by the feature value for each compound (red = high, blue = low). Features are ranked by their overall importance to model output.

SHAP (SHapley Additive exPlanations) analysis was conducted across all descriptors sets to uncover the internal decision-making logic of the best-performing QSAR models (Figure 4). This approach highlighted which molecular features had the most substantial influence on predictions and revealed how variations in these features shifted the predicted pIC50 values. Summary bar plots ranked descriptors by mean SHAP value, while dependence plots illustrated directional impacts, nonlinear behaviors, and interaction effects between features.

**Figure 5:**
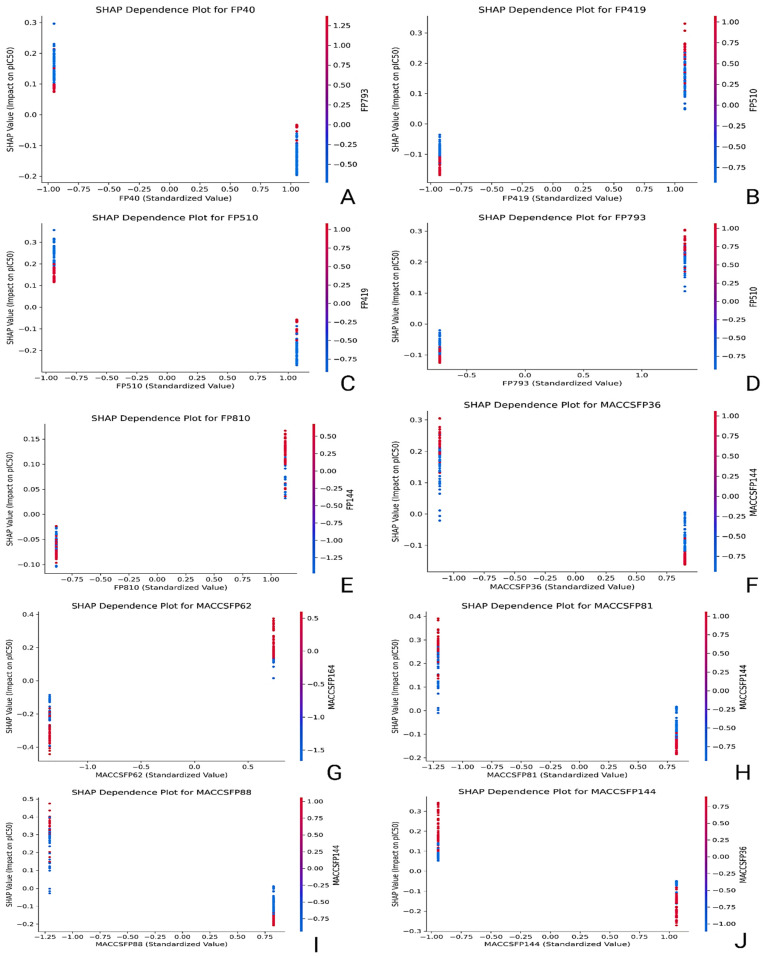
SHAP dependence plots showing the influence of top-ranked molecular descriptors on predicted pIC_50_. (**A**–**E**) CDK descriptors; (**F**–**J**) MACCS descriptors. The *x*-axis shows standardized descriptor values, and the *y*-axis shows SHAP values (impact on predicted pIC_50_). Each point represents a compound, colored by the value of a correlated descriptor (red = higher, blue = lower). Positive SHAP values increase predicted pIC_50_, and negative values decrease it; larger absolute SHAP values indicate stronger influence.

**Figure 6:**
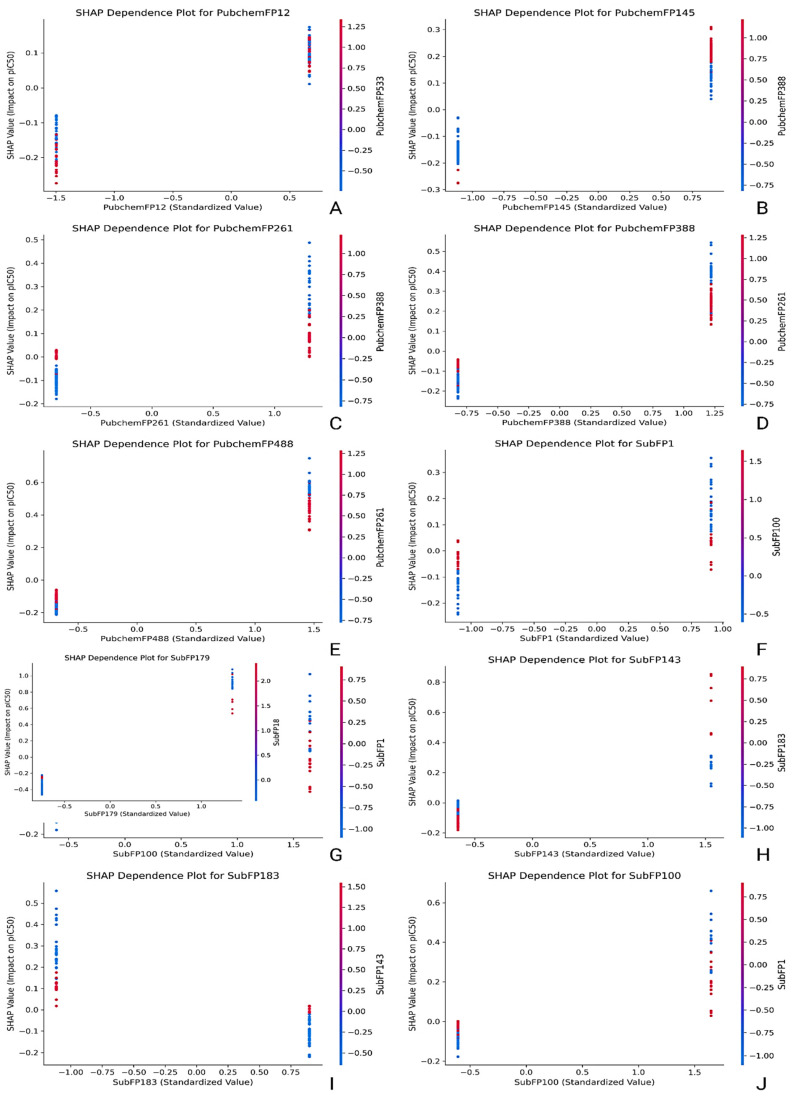
SHAP dependence plots showing the influence of top-ranked molecular descriptors on predicted pIC_50_. (**A**–**E**) PubChem descriptors; (**F**–**J**) substructure descriptors. The *x*-axis shows standardized descriptor values, and the *y*-axis shows SHAP values (impact on predicted pIC_50_). Each point represents a compound, colored by the value of a correlated descriptor (red = higher, blue = lower). Positive SHAP values increase predicted pIC_50_, and negative values decrease it; larger absolute SHAP values indicate stronger influence.

In the CDK-based model, FP510, FP419, FP793, FP40, and FP810 contributed the most to the model output. FP510 consistently increased predicted pIC50 values and exhibited the highest SHAP magnitude, suggesting it encodes a critical pharmacophoric element. Co-occurrence of FP793 and FP419 with FP510 amplified the model’s response, indicating synergistic interactions between these features. FP40 showed moderate influence, with a slightly nonlinear impact across its distribution. While the chemical meaning of CDK fingerprints could not be explicitly identified due to hashing, their stable predictive behavior and consistent ranking across samples validate their significance (Figure 5). The MACCS-based model offered interpretable insights through clearly defined fragment descriptors. MACCSFP62 (C–S bond) produced the highest SHAP values, aligning with known thiol-based interaction potential. MACCSFP88 (O–X linkage), MACCSFP81 (O–S bond), MACCSFP36 (C(NO)), and MACCSFP144 (N=I pattern) also positively impacted predictions. These features are often associated with electronegative atoms, hydrogen bond donors/acceptors, or rare but reactive motifs—each contributing to *LIMK1* binding. Their presence in influential positions underlines the relevance of oxygen- and sulfur-rich motifs in modulating activity and affinity (Figure 5). In the PubChem fingerprint-based model, the most significant descriptors included PubchemFP488 (N–C=N–H), FP388 (C(:C)(:N)(:N) motif), FP145 (≥1 nitrogen-containing five-membered ring), FP12 (≥16 carbon atoms), and FP261 (≥4 aromatic rings). PubchemFP488 had the strongest impact, with consistent elevation in pIC50 values when present. FP388 and FP145 showed sharp gradient shifts in dependence plots, indicating robust contributions from triazine-like and aromatic nitrogen moieties. FP12 and FP261 reflected overall size and aromaticity, both of which are common in kinase inhibitors. Together, these descriptors captured molecular bulk, planarity, and electronic characteristics crucial to *LIMK1* binding (Figure 6).

The substructure model offered the most interpretable and chemically grounded insights. SubFP179 (basic hetero nitrogen) emerged as the dominant contributor, with a steep SHAP curve supporting its association with increased biological activity. SubFP143 (carbonic acid derivative) and SubFP183 (sulphur-containing fragment) also contributed strongly, aligning with known features involved in hydrogen bonding and redox modulation. SubFP1 (primary carbon) and SubFP100 (secondary amide) showed consistent, additive influence, suggesting these motifs promote scaffold flexibility and contribute to critical polar contacts (Figure 6). The combined effect of these features was evident in their co-gradient behavior across dependence plots. Thus, the SHAP interpretation framework added a mechanistic dimension to the QSAR analysis. Substructure and MACCS descriptors provided high interpretability due to their explicit chemical definitions, making them well-suited for SAR-driven optimization. CDK features, despite being less transparent, displayed consistent model behavior and synergistic effects. PubChem fingerprints showed high variability but captured rich information related to aromaticity, heterocycles, and molecular size. The full report is compiled in the Supplementary Materials (Tables S23–S29, Figure S1). These insights will serve as a rational guide for future scaffold optimization and feature prioritization in the design of *LIMK1*-targeted therapeutics for stroke.

### 2.5. Compound-Level SHAP Attribution: Active vs. Inactive Profiles

**Figure 7:**
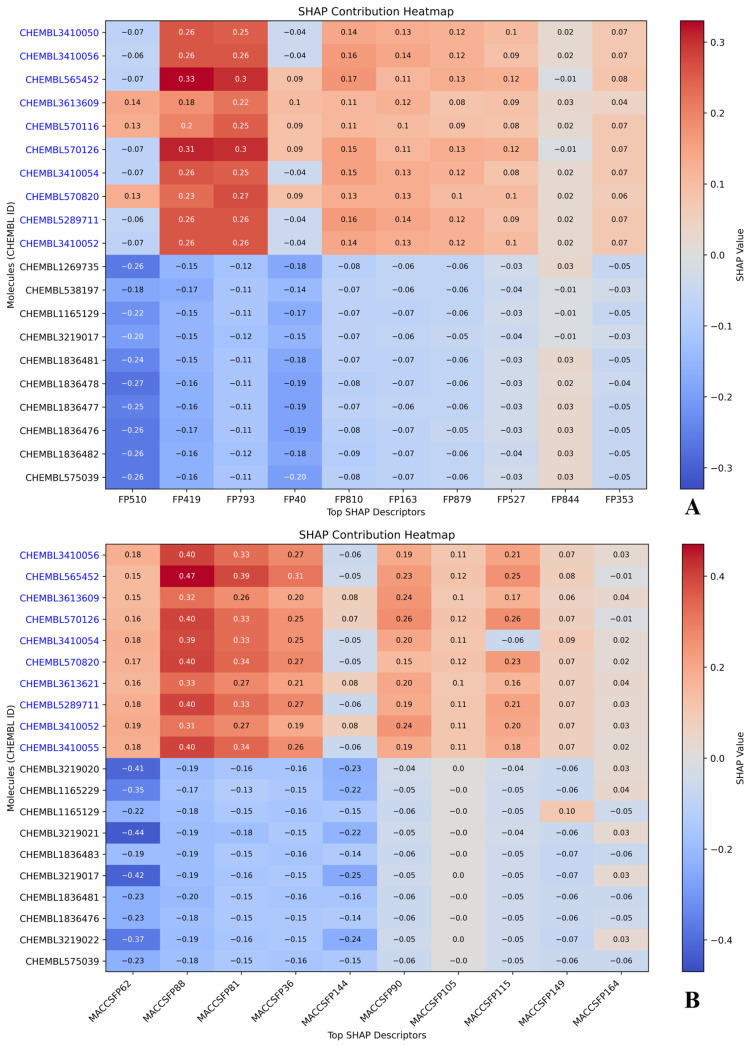
SHAP contribution heatmaps for top-ranked molecular descriptors across active (blue text) and inactive (black text) compounds. (**A**) CDK descriptors; (**B**) MACCS descriptors. Descriptor codes represent molecular fingerprint features encoding specific chemical substructures. The *x*-axis shows top SHAP-ranked descriptors, and the *y*-axis shows compound IDs. Warmer colors (red) indicate a stronger positive effect on predicted pIC_50_, and cooler colors (blue) indicate a stronger negative effect; color intensity reflects the absolute SHAP value, with larger values indicating stronger influence.

**Figure 8:**
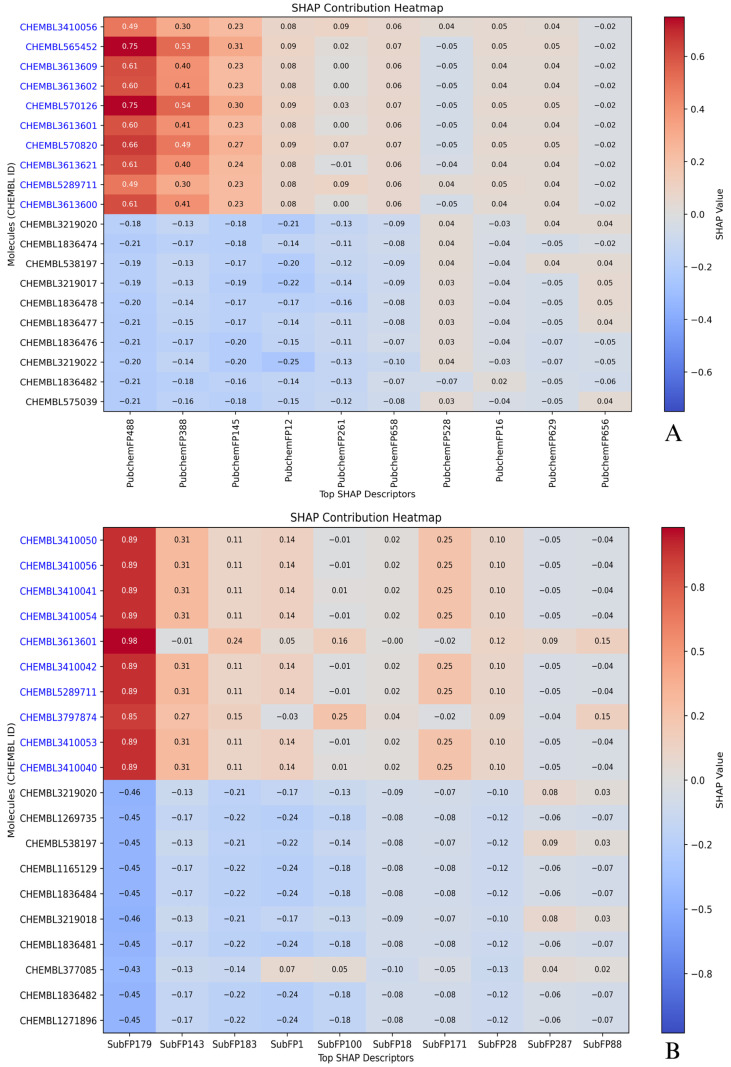
SHAP contribution heatmaps for top-ranked molecular descriptors across active (blue text) and inactive (black text) compounds. (**A**) PubChem descriptors; (**B**) substructure descriptors. Descriptor codes represent molecular fingerprint features encoding specific chemical substructures. The *x*-axis shows top SHAP-ranked descriptors, and the *y*-axis shows compound IDs. Warmer colors (red) indicate a stronger positive effect on predicted pIC_50_, and cooler colors (blue) indicate a stronger negative effect; color intensity reflects the absolute SHAP value, with larger values indicating stronger influence.

Compound-wise SHAP contribution heatmaps were generated using the top 10 most active and top 10 most inactive molecules from each descriptor-based model (Figure 7 and Figure 8). These visualizations enabled direct comparison of how influential each top-ranked descriptor was in driving the predicted pIC_50_ values for individual compounds. Positive SHAP values indicated an enhancing effect on predicted activity, while negative values reflected inhibitory contributions. Color gradients ranged from red (high positive influence) to blue (negative influence), highlighting contrast between highly active and poorly performing molecules. Several active compounds, such as CHEMBL3410050 and CHEMBL3410056, consistently appeared across all descriptor sets. In the CDK heatmap, these molecules showed strong positive SHAP values for descriptors like FP510, FP793, and FP419, which had already emerged as key predictors in the dependence plots. In MACCS, descriptors like MACCSFP62 (C–S bond) and MACCSFP88 (O–X linkage) contributed meaningfully to the same compounds, further strengthening the model’s internal agreement (Figure 7). These molecules also received elevated SHAP values from PubchemFP488 and FP388, which are associated with heteroatoms and aromatic systems, as well as from substructure features like SubFP179 and SubFP143, linked to basic nitrogen centers and carbonic acid derivatives. Another active compound, CHEMBL5289711, also exhibited high SHAP contributions across multiple descriptor sets. Its consistent placement near the upper right corner of each heatmap, especially under features such as SubFP179 and PubchemFP488, indicated strong agreement among descriptor classes in recognizing its pharmacophoric relevance (Figure 8). These repeated patterns support the prioritization of this compound for downstream experimental validation or analog synthesis.

On the inactive side, molecules like CHEMBL3219020 and CHEMBL538197 were frequently observed across heatmaps with uniformly negative SHAP contributions. Substructure-based descriptors such as SubFP143 and SubFP183 had a diminished or negative influence on these molecules. Likewise, in the MACCS and PubChem models, features with strong positive contributions in active compounds contributed negligibly or negatively here, supporting the classification as inactives. These patterns validate the model’s capacity to distinguish between functional and non-functional chemical scaffolds using consistent descriptor behavior. The shared presence of CHEMBL3410050, CHEMBL3410056, and CHEMBL5289711 among actives and CHEMBL3219020 and CHEMBL538197 among inactives across descriptor sets demonstrates descriptor-level redundancy working in favor of robust prediction. Rather than isolated reliance on one fingerprint, the model derived consistent prediction signals across substructure, CDK, MACCS, and PubChem, ensuring stability of interpretation. These findings add depth to the SHAP analysis by confirming that specific molecules not only possess relevant chemical features but are also consistently interpreted as such, regardless of fingerprint methodology. This compound-level SHAP profiling provides a valuable interpretability layer, reinforcing confidence in model-derived hits while offering clarity on inactive classification. Results from this analysis can guide structure prioritization and optimization in future *LIMK1*-targeted therapeutic design.

### 2.6. Molecular Docking Analysis

**Figure 9:**
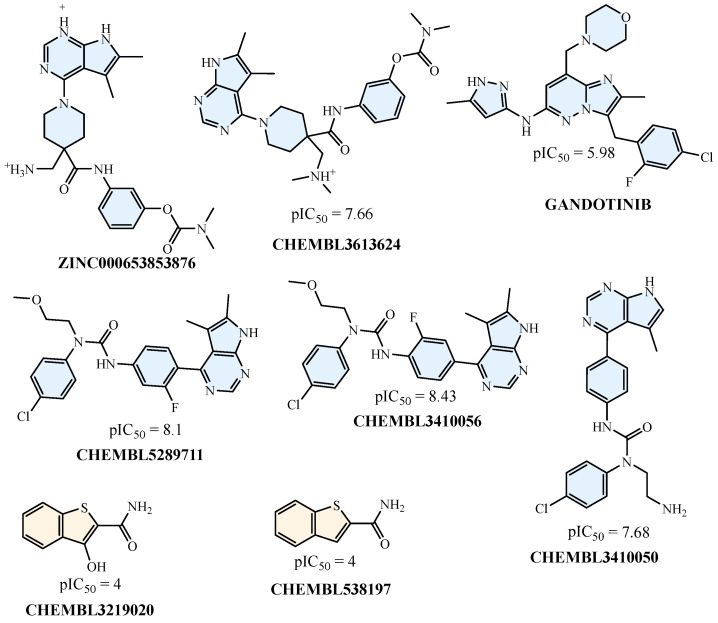
Structural representations of active compounds (blue) and inactive compounds (yellow).

**Figure 10:**
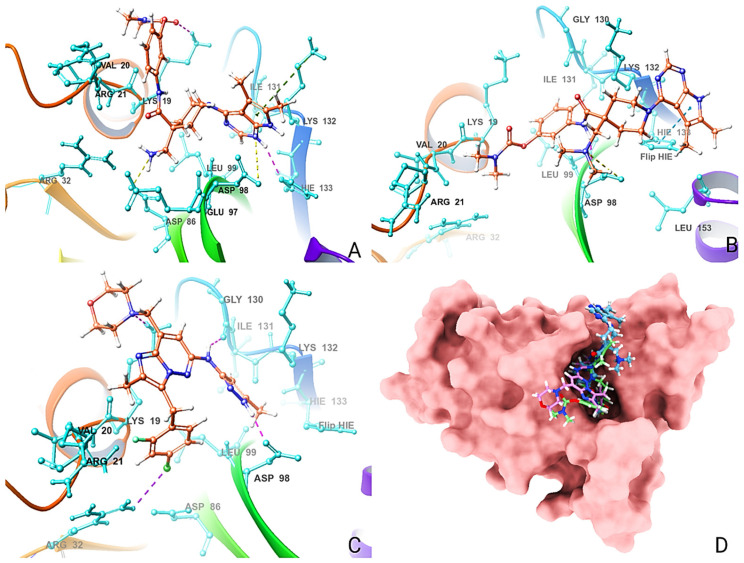
Protein–ligand interactions within the cofilin binding site. (**A**) Binding pose of CHEMBL3613624 showing key interactions at the active region. (**B**) Docking configuration of ZINC000653853876 within the cofilin pocket. (**C**) Interaction profile of Gandotinib at the target site. (**D**) Surface representation of cofilin showing the spatial alignment of all three ligands within the binding cavity.

Following QSAR modeling on known *LIMK1* inhibitors, top-ranking compounds and chemically similar analogs were evaluated through molecular docking to assess their repurposing potential as direct inhibitors of cofilin (Figure 9, Figures S2 and S3). Although *LIMK1* is upstream of cofilin in the signaling cascade, direct cofilin modulation offers a novel strategy for therapeutic intervention in stroke. Binding interactions were assessed, and interaction maps were generated for key hits using grid residues informed by *LIMK1*–cofilin structural studies [14]. Compounds were selected based on QSAR-predicted pIC_50_, SHAP-derived importance, and docking score thresholds, with a range spanning from −4.38 to −6.68 kcal/mol. CHEMBL3613624, the top-ranked hit with a docking score of −6.68 kcal/mol, demonstrated strong compatibility with the cofilin binding pocket. The compound formed a directional hydrogen bond with Asp98 and established π–π stacking with His133 through its pyrrolo[2,3-*d*]pyrimidine ring, a scaffold also central to its high QSAR-predicted activity. A salt bridge was formed with Asp98, further anchoring the molecule in the active site. Electrostatic complementarity was evidenced by the proximity of several positively charged residues (Lys19, Lys96, Lys132, Lys152, Arg21, Arg32), while Glu97 provided a negatively charged anchor. Hydrophobic contacts with Met18, Leu99, Ile131, Val20, and Leu153 supported structural stabilization, with flexible residues Gly130 and Gly154 offering dynamic accommodation (Figure 10). These combined interactions suggest that CHEMBL3613624 mimics essential pharmacophoric behavior originally evolved to bind *LIMK1* but can be effectively redirected toward cofilin. ZINC000653853876, identified via Tanimoto similarity from the ZINC database, showed a docking score of −6.25 kcal/mol. The molecule engaged His133 via hydrogen bonding through its pyrrolo[2,3-*d*]pyrimidine NH, and its carbonyl oxygen accepted a hydrogen bond from Lys19. Additional salt bridges with Asp86 and Asp98, alongside π–cation interaction from Lys132, suggested strong electrostatic fit. Hydrophobic residues including Ile131, Leu99, Val20, and Met18 surrounded the ligand, while polar contacts from Asn16 and charged residues such as Arg21 and Glu97 enriched the interaction profile. The compound’s binding behavior closely aligned with chemical space prioritized by SHAP-based model interpretation, particularly in its use of aromatic nitrogen-rich rings and electrostatic surface.

Gandotinib, a clinical candidate with a docking score of −5.61 kcal/mol, exhibited a diverse interaction network. Three hydrogen bonds were observed—Gly130 and Asp98 formed classic H-bonds with donor NH groups, while Lys19 interacted with a morpholine oxygen. Arg32 formed a halogen bond, enhancing specificity, and polar contacts with Thr129 and His133 suggested additional stability. Hydrophobic interactions were also prevalent, particularly with Leu99, Met18, Ile131, and Val20. The compound’s structural motif aligns with SHAP-identified features such as oxygen–nitrogen linkages and aromatic cores, further validating the QSAR-guided docking pipeline. Thus, these docking results support the viability of repurposing *LIMK1*-targeted chemotypes toward cofilin inhibition. Compounds such as CHEMBL3613624 and ZINC000653853876 exhibited favorable binding profiles with a convergence of hydrogen bonding, electrostatic anchoring, and hydrophobic compatibility. The consistent presence of Asp98, His133, Gly130, and Lys132 across active ligands points to a conserved pharmacophore region within the cofilin interface (Figure 10). Furthermore, these interaction profiles reinforce trends observed in SHAP and heatmap analyses, where certain substructures (such as pyrrolo–pyrimidine cores, basic nitrogen motifs) were highlighted as predictive of high activity.

### 2.7. Molecular Dynamics Simulation and MMGBSA

A 300 ns molecular dynamics simulation was performed for the top three ligand–cofilin complexes to evaluate their dynamic stability and intermolecular interactions. Compounds CHEMBL3613624, ZINC000653853876, and Gandotinib were selected based on prior docking, similarity, and clinical relevance. Each simulation was analyzed for structural deviations, flexibility, and interaction persistence using a combination of trajectory plots and contact frequency heatmaps.

**Figure 11:**
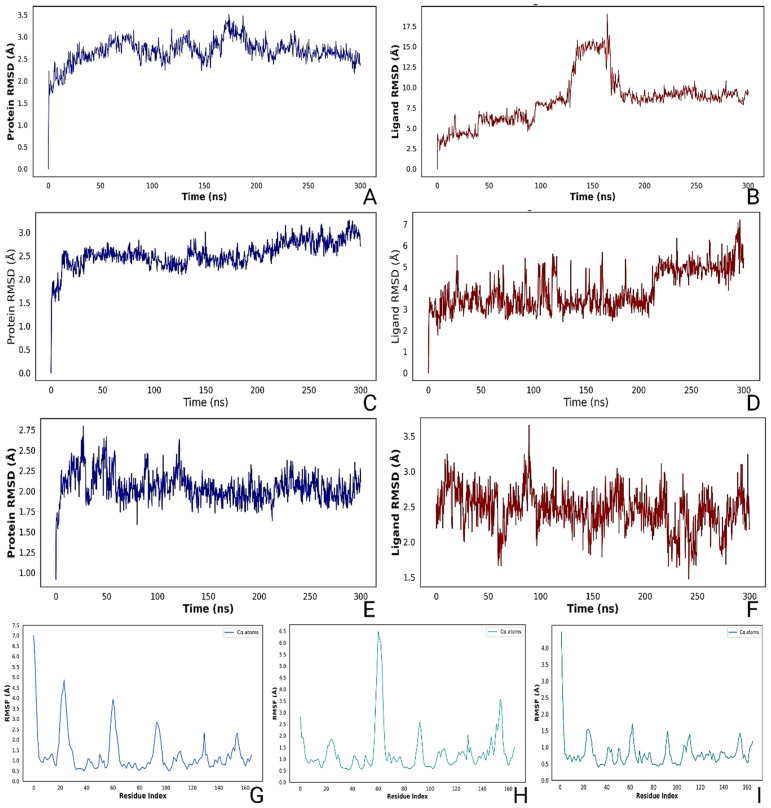
Molecular dynamics simulation analysis of top hit compounds. (**A**,**B**) RMSD plots of cofilin and CHEMBL3613624. (**C**,**D**) RMSD plots of cofilin and ZINC000653853876. (**E**,**F**) RMSD plots of cofilin and Gandotinib. (**G**–**I**) RMSF plots of cofilin in complex with CHEMBL3613624, ZINC000653853876, and Gandotinib, respectively.

CHEMBL3613624 initially entered equilibration around 25 ns and showed a brief high RMSD phase between 130 and 160 ns, peaking at 14 Å. After 170 ns, the ligand settled into a more stable state, maintaining ~7.6 Å RMSD until the end of the simulation. The mean protein–ligand RMSD over the trajectory was 2.70 ± 0.92 Å, with an average radius of gyration (rGyr) of 5.11 ± 0.38 Å, solvent-accessible surface area (SASA) of 350.77 ± 64.95 Å^2^, and polar surface area (PSA) of 111.26 ± 10.45 Å^2^ (Table S30). The protein backbone RMSD remained stable, fluctuating within 3.5 Å. RMSF analysis indicated notable fluctuations at residues near the N-terminus and loop regions, particularly around positions 20 and 60. Secondary structure analysis revealed 15.61% α-helix and 23.99% β-strand content, indicating moderate preservation of the global fold (Figure 11). Analysis of protein–ligand contacts revealed a persistent hydrogen bond between the ligand’s NH group and Asp98. Hydrophobic interactions were observed with Tyr89, His133, and Lys96, while Ser94 consistently mediated a water-bridged hydrogen bond. Frequent hydrogen bonds were observed with Asp98 (77.9%), supported by additional interactions with Glu134, Glu93, and Ser94. Water-mediated contacts involved residues like Thr129 and Gly154. Leu153 contributed significantly to hydrophobic interactions (28.2%), alongside Tyr89 (57.3%) and Leu99. The ligand also formed pi–pi stacking interactions with His133 (99.6%) and pi–cation contacts with Lys96 (110.5%) and Lys152. Intramolecular H-bonding increased gradually after 180 ns, indicating folding and structural tightening (Figure S4). The ligand’s radius of gyration and solvent-accessible surface area both decreased over time, suggesting increasing compactness and solvent burial. Fluctuations in PSA and MolSA aligned with structural rearrangements during equilibration and stabilization phases (Figure S5).

ZINC000653853876 showed equilibration by 32 ns and maintained two stability windows, first between 180 and 225 ns at ~3.1 Å RMSD and then from 245 to 265 ns near 4.5 Å. Protein RMSD remained consistent under 3 Å. The mean protein–ligand RMSD was 0.92 ± 0.38 Å, with rGyr of 4.67 ± 0.28 Å, SASA of 323.40 ± 27.00 Å^2^, and PSA of 162.01 ± 6.08 Å^2^ (Table S30). The complex retained 14.94% helix and 26.37% strand content, totaling 41.31% SSE. RMSF revealed peak fluctuations at residue 60, likely contributing to ligand-induced flexibility (Figure 11). Direct hydrogen bonding interactions were observed with Met18, Asp86, and Glu97. Additional bridging water molecules enabled interactions with Arg21 and Arg32, which linked to the carboxamide group of the ligand. Pi–cation interactions involving Arg21 and the benzene ring of the ligand further stabilized the complex. Thr129 and Lys19 contributed to water-mediated contacts, highlighting the cooperative role of polar residues in ligand positioning. The compound established strong hydrogen bonds with Met18 (97.6%), Asp86 (99.7%), and Glu97 (116.9%). Gly130 also contributed with 54.5% H-bond occupancy. Water-bridged contacts occurred with Arg21, Arg32, Thr129, and Lys19, while pi–cation interactions were observed with Arg21 (89.4%) and Lys19 (46.3%). Pi–pi stacking was exclusive to Phe15 (100%) (Figure S4). The ligand exhibited consistent intramolecular H-bonding early in the trajectory but became more flexible after 250 ns. RMSD, MolSA, SASA, and PSA values displayed modest fluctuation, with a sharp drop in radius of gyration after 240 ns. These transitions reflect a conformation shift that likely enhanced fitting into the binding cleft (Figure S6).

Gandotinib reached equilibration near 30 ns and maintained exceptional stability between 150 and 230 ns at 2.65 Å and again from 280 to 300 ns at 2.70 Å. The protein RMSD was the lowest among all complexes, stabilizing under 2.75 Å. The mean protein–ligand RMSD was 1.55 ± 0.19 Å, with rGyr of 4.67 ± 0.07 Å, SASA of 210.07 ± 24.17 Å^2^, and PSA of 94.76 ± 5.24 Å^2^ (Table S30). The system displayed 16.58% helix and 27.32% strand content, yielding the highest SSE at 43.90%. RMSF analysis confirmed minimal flexibility across the trajectory. Hydrogen bonds were formed between the NH group of the pyrazole ring and Asp98, while Gly130 interacted with the linker NH group. Additionally, Glu97 formed a water-bridged hydrogen bond with the ligand, contributing to structural anchoring (Figure 11). Pi–cation interactions were observed between Arg32 and the benzene ring, and between Lys132 and the pyrazole, further reinforcing the electrostatic fit. Leu99 maintained a hydrophobic contact, aligning with ligand burial in the binding pocket. Hydrogen bonding occurred primarily with Gly130 (99.6%) and Asp98 (96.4%). The ligand displayed minimal water bridges but interacted indirectly with Glu97 via solvent molecules (Figure S4). Hydrophobic contact was maintained with Leu99 (94.0%), and pi–cation interactions occurred with Arg32 (51.0%) and Lys132 (86.8%). Gandotinib exhibited the most consistent PSA and MolSA trajectories, and no intramolecular hydrogen bonding was observed throughout the simulation. Its compact radius of gyration and narrow SASA range pointed to a tightly folded structure with deep insertion into the protein pocket (Figure S7).

Among the three candidates, CHEMBL3613624 demonstrated the highest interaction diversity, engaging polar, charged, and hydrophobic residues along with extensive pi stacking. However, its RMSD fluctuation suggested a late-stage structural rearrangement. ZINC000653853876 provided a stable mid-range profile with strong polar anchoring and dynamic reorganization after 250 ns. Gandotinib offered the most stable conformation with persistent interactions, low flexibility, and minimal energetic drift, making it a promising cofilin binder under physiological conditions.

### 2.8. Energetic and Conformational Insights from MM-GBSA, DCCM, and PCA Analyses

**Figure 12:**
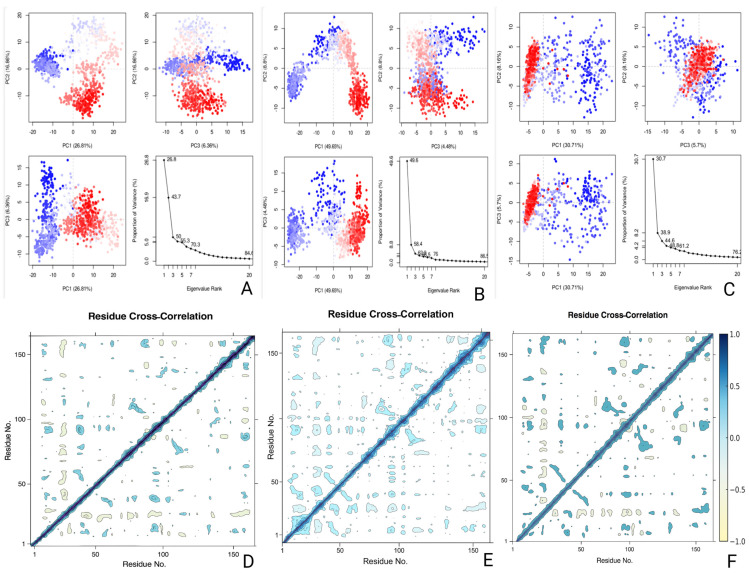
Principal component analysis (PCA) and residue cross-correlation (DCCM) of cofilin bound with CHEMBL3613624 (**A**,**D**), ZINC000653853876 (**B**,**E**), and Gandotinib (**C**,**F**). PCA was performed using the Cα coordinates from MD simulation trajectory files generated over 300 ns and analyzed with the Bio3D package in R v2.4-5. In the PCA plots, dark blue indicates the highest mobility, light blue and red indicates intermediate mobility, and dark red the lowest mobility. DCCM maps illustrate correlated and anti-correlated residue motions during a 300 ns MD simulation.

Molecular dynamics simulations were further evaluated using MM-GBSA binding energy profiles, principal component analysis (PCA), and dynamic cross-correlation matrices (DCCM) to understand the energetic favorability and collective motion patterns induced by ligand binding (Figure 12). These methods provided a deeper understanding of stability and flexibility across the cofilin complexes with CHEMBL3613624, ZINC000653853876, and Gandotinib. MM-GBSA calculations were performed at 0, 100, 200, and 300 ns to assess temporal variation in ligand–cofilin binding energy. ZINC000653853876 consistently showed the most favorable ΔG_bind values across all frames, including −77.80 kcal/mol at 100 ns and −77.21 kcal/mol at 300 ns. These values were mainly driven by strong Coulombic interactions (–42.05 kcal/mol at 100 ns) and significant hydrophobic contributions (−25.31 kcal/mol at 300 ns). Gandotinib recorded its strongest binding energy at 200 ns (−77.16 kcal/mol), supported by large lipophilic energy (−30.26 kcal/mol) and Coulombic terms (−18.26 kcal/mol). For CHEMBL3613624, the best ΔG_bind was observed at 0 ns (−62.79 kcal/mol) and remained stable at −61.98 kcal/mol at 300 ns (Table S31). Its binding profile involved balanced electrostatic and lipophilic contributions, with no extreme peaks. None of the three compounds showed large covalent energy or packing penalties, and solvation terms varied modestly without destabilizing effects.

Principal component analysis revealed the extent and diversity of global motions. CHEMBL3613624 displayed moderate flexibility, where PC1 accounted for 26.8% of variance, PC2 for 16.86%, and PC3 for 6.36%. Its eigenvalue curve showed a gradual decline, indicating distribution. ZINC000653853876 exhibited the highest PC1 contribution at 49.65%, suggesting one dominant directional shift during simulation. PC2 and PC3 explained 8.8% and 4.48%, respectively. Gandotinib showed PC1 variance of 30.71%, followed by 8.16% (PC2) and 5.7% (PC3). Its eigenvalue profile dropped sharply after the first two components, which reflected constrained movement. PCA scatter plots confirmed that Gandotinib occupied a compact conformational space, while CHEMBL3613624 and ZINC000653853876 covered a broader range of structures over time (Figure 12). Cross-correlation matrices provided insights into how residue pairs fluctuated during the simulation. For CHEMBL3613624, positively correlated regions were observed along the diagonal, particularly in central secondary structure blocks. Weak anti-correlations emerged between flexible loop segments and distal sites, especially around residues 60–90, consistent with observed RMSF fluctuations. ZINC000653853876 induced stronger off-diagonal correlation patterns, especially between residues 30–50 and 140–160 (Figure 12). These patterns reflected cooperative movement of distant residue clusters, likely associated with ligand anchoring through polar contacts. Gandotinib produced sparse correlation features, with most values near zero. Only a few positive correlations were seen between core residues, and negative fluctuations were minimal. This indicated that the ligand remained stable without triggering large internal motion shifts in the protein.

The DCCM also reflected how ligand binding influenced structural coupling. Residues with positive correlations moved in similar directional trends, while negatively correlated pairs fluctuated in opposite directions. CHEMBL3613624 induced modest coordinated behavior, with loop and turn regions contributing to most of the fluctuation. ZINC000653853876 introduced broader dynamic regions, including charged and polar residues, consistent with its hydrogen bonding and water bridge profiles. Gandotinib, by contrast, maintained minimal internal perturbation, which aligned with its low RMSF, stable PC distribution, and compact MM-GBSA energy fluctuations. Thus, the results confirmed that ZINC000653853876 exhibited the strongest energetic interaction profile, particularly through Coulombic and hydrophobic contributions, while Gandotinib maintained the most structurally stable and compact profile with limited flexibility (Figure 12). CHEMBL3613624 showed diverse motions and a broad range of interactions, albeit with slightly less favorable binding energy. Each ligand influenced cofilin conformation in distinct ways, highlighting the benefit of integrating energetic and dynamic descriptors when evaluating drug repurposing candidates. Overall, CHEMBL3613624 and ZINC000653853876 promoted broader conformational sampling and more extensive correlated motions compared to Gandotinib, suggesting greater potential to modulate dynamic regions critical for target function.

### 2.9. Network Pharmacology-Based Functional Mapping of Hit Compounds in the Stroke Context

**Figure 13:**
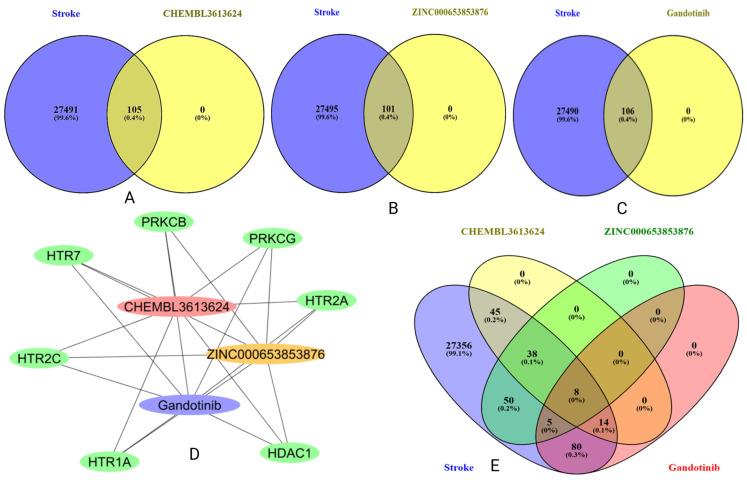
Gene overlap and network analysis of repurposed compounds with stroke-related targets. Venn diagrams (**A**–**C**) show common genes between stroke and each compound: CHEMBL3613624 (**A**), ZINC000653853876 (**B**), and Gandotinib (**C**). (**D**) Gene–compound network displays eight shared targets. (**E**) A Venn diagram highlights the eight common genes shared across all three compounds and stroke, suggesting shared mechanistic relevance.

A network pharmacology framework was adopted to extend the molecular findings and assess whether the top repurposed compounds could modulate stroke-related biological mechanisms beyond cofilin binding. Targets linked to CHEMBL3613624, ZINC000653853876, and Gandotinib were collected from public databases and systematically compared with stroke-associated genes. The intersection revealed eight overlapping genes, *MAPK1*, *PRKCB*, *HDAC1*, *HTR2A*, *HTR1A*, *PRKCG*, *HTR7*, and *HTR2C*, which were common to all three compounds and the stroke gene set (Figure 13; Table 3, Tables S32 and S33). This convergence suggests that these structurally diverse molecules may exert similar therapeutic effects by acting on shared nodes implicated in stroke pathophysiology. Protein–protein interaction (PPI) analysis using the STRING database provided deeper insight into the functional architecture of these targets. The resulting network was organized into three functional clusters. The first group consisted of serotonin receptor genes, including *HTR1A*, *HTR2A*, *HTR2C*, and *HTR7*, which are involved in cerebrovascular regulation, mood modulation, and synaptic transmission. The second cluster featured intracellular signaling kinases *MAPK1*, *PRKCB*, and *PRKCG*, known for their roles in inflammatory cascades, neuronal death, and blood–brain barrier integrity following ischemic events. The third cluster included *HDAC1*, an epigenetic regulator involved in chromatin remodeling and DNA repair processes that are activated in response to ischemic injury [15,16].

Gene–compound network mapping showed that CHEMBL3613624 was connected to all eight stroke-relevant targets, indicating the broadest interaction profile. ZINC000653853876 showed overlap with six targets, while Gandotinib shared five, most notably including multiple serotonin receptors and *HDAC1*. These differences highlight varying degrees of pharmacological coverage, with CHEMBL3613624 standing out as the most versatile candidate for further development. Functional enrichment analysis of these common genes revealed high-confidence involvement in several stroke-associated biological pathways (Figure S8 and Tables S34–S38). Notably, serotonergic synapse signaling was enriched due to the presence of *HTR1A*, *HTR2A*, *HTR2C*, and *HTR7*. Additional enrichment of *MAPK* signaling and *PKC*-related pathways further supported their role in neuroinflammatory and apoptotic regulation [17,18]. *HDAC1*-driven pathways related to chromatin modification and gene expression recovery were also significantly represented. The enrichment analysis demonstrated strong statistical confidence, with multiple pathways showing false discovery rates (FDRs) well below 0.0001.

Published literature supports the involvement of these genes in stroke biology. *MAPK1* and the *PKC* isoforms *PRKCB* and *PRKCG* have been shown to mediate pro-apoptotic responses, oxidative stress, and neuroinflammation following cerebral ischemia [19,20]. *HDAC1* contributes to axonal regeneration and neuronal survival post-stroke, often through epigenetic mechanisms. Serotonin receptors such as *HTR2A* and *HTR7* are associated with vasodilation, platelet aggregation, and cognitive recovery and have been explored as therapeutic targets for ischemic and post-stroke depression [21,22]. By connecting ligand-level interactions with gene-level regulatory networks, this analysis confirms that the top candidate compounds not only show stable and favorable dynamics at the cofilin target but also overlap with key pathways known to contribute to stroke onset, progression, and recovery. This systems-level insight adds translational value to the earlier computational predictions and provides a mechanistic rationale for prioritizing these compounds in future experimental studies.

## 3. Discussion

Cofilin plays a central role in actin cytoskeletal remodeling, and its dysregulation is associated with critical events in stroke, such as neuronal injury, inflammation, and disruption of the blood–brain barrier [23,24,25]. Despite this relevance, direct targeting strategies for cofilin remain underexplored. This study aimed to repurpose *LIMK1* inhibitors originally designed to regulate cofilin through phosphorylation by evaluating their potential to bind directly to cofilin. The objective was to build a comprehensive cheminformatics and network-based workflow to identify, validate, and contextualize new cofilin modulators within the scope of ischemic stroke. The study began by creating QSAR models trained on 204 *LIMK1* inhibitors using four types of molecular fingerprints. Instead of focusing only on numerical performance, the analysis emphasized chemical interpretability. Descriptors related to sulfur linkages, nitrogen heterocycles, and polar functionalities were consistently associated with high activity, suggesting their relevance to both kinase interaction and possible cross-reactivity with actin-regulating proteins like cofilin. Models such as MACCS–KNN and CDK–Ridge Regression not only showed good statistical performance but also highlighted key molecular traits. These findings guided the prioritization of compounds for further structural studies.

SHAP analysis provided additional insight by clarifying which molecular fragments contributed most to activity predictions. Basic nitrogen groups, aromatic systems, and sulfur-containing moieties were consistently linked with higher predicted potency. These fragments aligned well with pharmacophoric features expected to interact with cofilin’s charged surface residues. This interpretability helped ensure that compound selection was driven by more than just performance metrics, creating a logical path toward docking analysis. Docking was used to examine whether high-ranking QSAR compounds could interact favorably with the cofilin interface, particularly residues such as Asp98, His133, and Gly130, which are known to influence cofilin–actin binding. The top compounds, CHEMBL3613624, ZINC000653853876, and Gandotinib, demonstrated strong binding affinities and formed stable interactions with several key residues. These included hydrogen bonds, salt bridges, and pi–cation interactions. Notably, many of the residues involved in docking were also implicated by SHAP descriptors, supporting a consistent link between molecular features and receptor binding patterns. Molecular dynamics simulations allowed us to assess how these interactions evolved over time. Gandotinib showed the most stable conformational profile, maintaining a narrow RMSD range and consistent contacts. CHEMBL3613624 initially showed flexibility but stabilized after 170 ns while maintaining a broad network of interactions. ZINC000653853876 exhibited two stability phases, indicating conformational adaptability. The chemical descriptors identified by SHAP were found to align closely with docking-derived pharmacophoric interactions. Basic hetero-nitrogen fragments (SubFP179, PubChemFP488, FP388) and aromatic nitrogen-rich rings (PubChemFP145, FP261) explained the strong directional hydrogen bonds and π–π stacking of CHEMBL3613624 with Asp98 and His133, further stabilized by salt bridges with Lys19 and Lys132. These contacts were not only predicted but also persisted during MD simulations, with Asp98 hydrogen bonding observed in ~78% of frames and His133 π–π stacking exceeding 99% occupancy. Similarly, oxygen–sulfur and oxygen–halogen linkages (MACCSFP81, FP88, FP144) correlated with Gandotinib’s interaction pattern, where Lys19 and Arg32 provided electrostatic anchoring, supported by high MD occupancies (Asp98 H-bond ~96%, Gly130 ~99%). For ZINC000653853876, descriptors such as secondary amides (SubFP100) and nitrogen-rich aromatic scaffolds (PubChemFP145, FP388) matched the strong hydrogen bonding to Asp86 and Glu97 and π-cation contacts with Arg21 and Lys19, which were consistently retained in simulations (Asp86 > 99%, Glu97 ~97%). Across all ligands, Asp98, Gly130, His133, and Lys132 emerged as conserved hotspots that reinforced the biological significance of descriptor-selected motifs. Thus, SHAP analysis, docking, and MD validation converged to highlight a coherent chemical–biological rationale for cofilin binding (Table S39). These simulations also confirmed that key interactions observed during docking, such as hydrogen bonding with Asp98 and pi–cation contact with His133 or Lys residues, persisted under physiological conditions.

Energy decomposition using MM-GBSA supported the stability observations. ZINC000653853876 showed the strongest overall binding energy, particularly due to strong electrostatic and lipophilic contributions. Gandotinib reached its best energy profile at 200 ns, matching the time frame of its lowest structural fluctuation. CHEMBL3613624 displayed consistent energy values throughout the trajectory, though slightly less favorable than the other two. These findings helped correlate kinetic behavior with energetic feasibility. Additional insights were gained from PCA and DCCM analyses. ZINC000653853876 explored broader conformational space, consistent with its dual-phase dynamics. Gandotinib remained tightly clustered, suggesting limited flexibility. CHEMBL3613624 showed moderate movement, which corresponded with its broad interaction network. DCCM patterns revealed that each ligand induced distinct residue correlation profiles. CHEMBL3613624 affected loop dynamics, ZINC000653853876 triggered cooperative movement in polar clusters, and Gandotinib caused minimal disturbance. These variations suggest that each compound influences protein motion differently, which could affect downstream signaling or functional outcomes. While these results highlight the promise of direct cofilin modulation, it is important to acknowledge that cofilin is a central regulator of actin filament turnover and cytoskeletal dynamics [26]. Altering its activity, whether through inhibition or stabilization, could potentially affect essential cellular processes, including vesicle trafficking, cell motility, and synaptic remodeling. Such off-target consequences may lead to unintended effects in non-neuronal tissues or in physiological contexts beyond ischemic injury [9,27]. Future studies should therefore assess selectivity profiles and cellular impacts in multiple models to ensure that the therapeutic benefits outweigh potential cytoskeletal disruptions.

To connect molecular findings with disease relevance, network pharmacology was used to map compound-associated genes against stroke-linked targets. Eight common genes were found across all three compounds and stroke: *MAPK1*, *PRKCB*, *PRKCG*, *HDAC1*, *HTR1A*, *HTR2A*, *HTR2C*, and *HTR7*. These genes formed three clusters related to serotonin signaling, kinase activity, and epigenetic regulation. Each of these processes is known to play a role in ischemic injury. For example, *MAPK1* and *PKC* isoforms contribute to inflammation and cell death, *HDAC1* influences neuronal repair, and serotonin receptors affect vascular function and synaptic plasticity [15,17,18,20,21].

Gene–compound mapping revealed that CHEMBL3613624 was connected to all eight targets, suggesting a broader pharmacological profile. ZINC000653853876 and Gandotinib shared fewer targets but were more selective. These differences may guide future decisions on compound optimization. CHEMBL3613624 could serve as a polypharmacological lead, Gandotinib as a stable binder with specific downstream effects, and ZINC000653853876 as an energetically favorable molecule with dynamic flexibility. What sets this work apart is the integration of structure-based, dynamic, and systems-level analyses. Rather than treating each step in isolation, the workflow maintained a logical link from descriptor-level predictions to protein-level interactions and gene-level relevance. SHAP-derived features aligned with docking hotspots, and dynamic behaviors reflected predicted energetic profiles. The gene networks further reinforced these connections by mapping them to known stroke biology. This alignment across computational levels enhances the confidence in these compounds as real candidates for therapeutic development. The findings not only support cofilin as a viable target for stroke but also provide a structured pipeline for future repurposing studies. 

While the integrated approach offers valuable mechanistic insights, there are some limitations to consider. The study relies entirely on predictive models, including QSAR, docking, molecular dynamics, and pathway mapping, without experimental validation at this stage. Docking has its own limits, such as using simplified protein structures, not fully considering solvent effects, and the risk of false positives due to scoring function errors. We used molecular dynamics and MMGBSA to improve reliability, but real binding experiments are needed to confirm the results. Binding affinity and stability predictions may not fully capture biological complexity, such as metabolic transformation, cellular uptake, or off-target effects. Additionally, the selection of stroke-related genes was based on available databases, which may not represent the complete disease network or context-specific expression patterns. Despite these limitations, the study contributes important early-stage evidence and offers a mechanistic rationale to support compound prioritization. The computational results provide a roadmap for wet-lab screening and help reduce the trial-and-error phase of drug development. This pipeline not only narrows down viable candidates but also highlights key structural and pathway-based interactions for future lead optimization. Furthermore, ischemic stroke is a heterogeneous condition influenced by factors such as age, comorbidities, and genetic background, which can lead to variability in patient responses to therapy. In vitro and in vivo studies across diverse models will be essential to address these complexities and confirm the translational potential of the identified compounds. This work sets the stage for translating computational findings into experimental insights for cofilin-modulating therapies in stroke.

## 4. Materials and Methods

### 4.1. Ligand Dataset Preparation

A dataset of small-molecule inhibitors targeting LIM domain kinase 1 (*LIMK1*; ChEMBL Target ID: CHEMBL3836, UniProt ID: P53667) was obtained from the ChEMBL database v2023 [28]. From an initial pool of 508 compounds associated with experimentally determined IC_50_ values, compounds with missing or non-numeric IC_50_ values and duplicated entries were removed. This curation process resulted in a final set of 312 unique molecules, each associated with a valid canonical SMILES string (Tables S40 and S41). To standardize the activity range, IC_50_ values were converted into their negative logarithmic scale (pIC_50_) using the formula –log_10_(IC_50_ [in molar units]). Based on the distribution of pIC_50_ values, compounds were classified into three categories: active (pIC_50_ ≥ 7), intermediate (6.0 ≤ pIC_50_ < 7.0), and inactive (pIC_50_ < 6.0). A total of 204 compounds belonged to either the active or inactive classes and were selected for the development of classification and regression models (Table S42). The remaining 108 compounds were designated as intermediates and excluded from modeling tasks to reduce boundary bias and improve class separability. All molecular structures were standardized prior to descriptor generation, and SMILES were retained for downstream cheminformatics analysis.

### 4.2. Molecular Descriptor Calculation

Molecular descriptors were generated using the PaDEL-Descriptor software v2.21 [29] through an automated Python v3.12.0 wrapper script employing the padelpy module. Each compound’s SMILES representation was used as input to compute a range of structural and fingerprint-based descriptors. Four distinct descriptor sets were curated to capture a broad representation of chemical features: MACCS keys (166-bit substructure patterns), PubChem fingerprints (881-bit), substructure fingerprints (307-bit), and CDK fingerprints (1024-bit) provided by the Chemistry Development Kit. Descriptor calculation was performed under default PaDEL parameters, ensuring consistent bit length and fingerprint encoding across all molecules. Each descriptor set was processed separately to evaluate its individual contribution to model performance. Molecules with missing descriptor values or computational errors during feature extraction were excluded to preserve data quality. The final curated descriptor matrices were used for QSAR modeling and subsequent machine learning analysis.

### 4.3. Feature Selection and Data Splitting

Before model development, low-variance features were removed using a Variance Threshold filter from the sklearn.feature_selection module. A cutoff value of 0.1 was applied, and descriptors with a variance below this threshold were discarded to eliminate uninformative features and reduce model complexity. The resulting descriptor matrices for each fingerprint set were then used for dataset partitioning. A stratified train–test split was performed using the train_test_split function from the sklearn.model_selection module, ensuring consistent class distribution across both training and testing subsets. The data were divided into an 80:20 ratio with a fixed random seed (random_state = 42) to maintain reproducibility. The training set was subsequently used for model construction, while the test set was reserved exclusively for external evaluation.

### 4.4. QSAR Model Development and Evaluation

Multiple regression algorithms were developed and evaluated using Python-based machine learning workflows executed in Google Colab (https://colab.google/). The algorithms applied included Random Forest, Support Vector Regression (SVR), Gradient Boosting, K-Nearest Neighbors (KNN), Bagging, Ridge Regression (Pace-like variant), Partial Least Squares (PLS), and Gaussian Process Regression. Model development was carried out using scikit-learn 1.6.1, pandas 2.2.2, numpy 2.0.2, and shap 0.48.0. Model development was carried out using the scikit-learn library, with algorithm-specific hyperparameters tuned where necessary. The final model configurations were as follows: Random Forest (n_estimators = 200, min_samples_split = 5, min_samples_leaf = 2, random_state = 42), SVR (kernel = ‘rbf’, C = 1.0, epsilon = 0.5), Gradient Boosting (n_estimators = 100, random_state = 42), KNN (n_neighbors = 5, Euclidean distance), Bagging (n_estimators = 100, random_state = 42), Ridge Regression (alpha = 1.0), PLS regression (n_components = 11), and Gaussian Process Regression (default scikit-learn parameters). For each descriptor set, models were trained using the curated training set, and predictions were evaluated on an external test set. The performance of each model was assessed using both regression metrics, such as Pearson correlation coefficient (R), coefficient of determination (R^2^), adjusted R^2^, root mean squared error (RMSE), and mean absolute error (MAE), and classification-based metrics, including precision, recall, specificity, and F1 score. For binary classification evaluation, compounds were assigned activity labels based on predefined pIC_50_ thresholds. The most predictive model from each descriptor category was further subjected to 5-fold cross-validation to assess generalizability. Models demonstrating superior performance across both validation and test datasets were retained for interpretation and compound prioritization.

### 4.5. Model Interpretation and Compound Prioritization

To interpret the developed QSAR models and guide compound prioritization, SHAP (SHapley Additive exPlanations) [30] analysis was performed on the top regression models. SHAP summary and dependence plots highlighted key molecular descriptors influencing activity predictions, enabling rational hit selection. Model applicability domains were assessed using Williams plots to detect outliers and influential data points based on leverage and residual analysis [31,32]. Only compounds within the defined boundaries were retained for further steps. Principal component analysis (PCA) was also applied to visualize chemical space coverage and descriptor diversity. These integrated interpretations informed the final selection of structurally relevant and predictive compounds for molecular docking.

### 4.6. Molecular Docking Studies

The crystal structure of human cofilin-1 (PDB ID: 4BEX) [14] was used for molecular docking to evaluate the binding potential of *LIMK1* inhibitor-derived compounds. Protein preparation was carried out using Schrödinger’s Protein Preparation Wizard v2021-2, where bond orders were assigned, missing side chains and hydrogen atoms were added, and the structure was minimized using the OPLS4 force field [33,34]. Crystallographic water molecules beyond 5 Å from hetero groups were removed to avoid non-specific interactions. Based on structural evidence from Klejnot et al. (2013) [14], His133—a residue functionally implicated in actin regulation—was selected as the grid center for docking, ensuring spatial coverage of the key binding pocket. Ligand structures from the QSAR-predicted active class were energy-minimized and prepared using LigPrep, and docking was performed using the Glide XP scoring function [33]. To expand the compound space, the top-performing hits were used as queries for Tanimoto similarity-based virtual screening against the ZINC database [35]. Molecules sharing ≥ 0.4 fingerprint similarity were retrieved and subjected to the same ligand preparation and docking workflow against the 4BEX structure. Additionally, a set of clinically approved or investigational drugs including those listed under various clinical phases were also docked as reference ligands to assess comparative binding affinity and interaction profiles.

### 4.7. Molecular Dynamics Simulations

Molecular dynamics (MD) simulations were performed to evaluate the dynamic stability and interaction persistence of the top docked compounds within the cofilin-1 binding site. The simulations were conducted using the Desmond module v2019-4 [36], applying the OPLS4 force field for all components. Protein–ligand complexes were embedded in an orthorhombic simulation box with periodic boundary conditions and solvated using the TIP3P water model [37]. Counterions (Na^+^/Cl^−^) were added to neutralize the system, and 0.15 M NaCl was included to mimic physiological ionic strength. The systems underwent initial energy minimization followed by an equilibration phase using the default Desmond relaxation protocol. Production runs were carried out for 300 ns under the NPT ensemble at 300 K and 1 atm using the Nose–Hoover thermostat and Martyna–Tobias–Klein barostat [38]. Trajectories were saved every 300 ps for analysis. Post-simulation analyses were performed. Additionally, MMGBSA, principal component analysis (PCA), and dynamic cross-correlation matrix (DCCM) calculations were used to investigate global motion patterns and residue communication dynamics [39].

### 4.8. Systematic Network Pharmacology

Putative gene targets for CHEMBL3613624, ZINC000653853876, and Gandotinib were identified using SwissTargetPrediction v2019 [40] and Superpred v3.0 [41] based on their SMILES representations. Only targets with high probability scores and relevance to human proteins were selected. Stroke-associated genes were retrieved from GeneCards [42], DisGeNET [43], and CTD databases [44], filtered using disease relevance scores to ensure ischemic stroke specificity. Gene overlap between each compound and stroke was analyzed using Venn diagrams v2.1.0. Eight genes common to all three compounds and stroke were selected for further exploration. These genes were analyzed through STRING v12.0 [45] for protein–protein interaction mapping, with a confidence score threshold of 0.7. Network visualization and clustering were performed in Cytoscape v3.9.1 [46]. Functional enrichment was conducted using STRING, referencing KEGG, GO Biological Process, Reactome, and WikiPathways [47,48]. Enrichment terms with FDR < 0.05 were considered significant. Visual summaries of enriched pathways and gene clusters were generated using STRING’s enrichment interface and Cytoscape plugins.

## 5. Conclusions

This study presents a comprehensive computational framework to identify and repurpose *LIMK1* inhibitors for direct targeting of cofilin in stroke therapy. Eight machine learning algorithms were evaluated across four descriptor sets (MACCS, substructure, CDK, and PubChem), with model selection based on balanced regression and classification performance. The best results were achieved with MACCS–Bagging, CDK–Ridge Regression, and substructure–Gradient Boosting, each offering strong predictability and interpretability. QSAR-derived hits were validated through molecular docking against cofilin’s active region, identifying CHEMBL3613624, ZINC000653853876, and Gandotinib as promising binders. These compounds were further investigated using 300 ns molecular dynamics simulations, which confirmed their stable conformational behavior and sustained binding interactions. MM-GBSA energy calculations and motion analyses, such as PCA and DCCM, provided additional support, highlighting ZINC000653853876 as the most energetically favorable compound and Gandotinib as the most conformationally stable. This study also explored how these compounds may work at the biological level. A network pharmacology analysis found eight genes shared between the compounds and known stroke targets. Network pharmacology analysis revealed eight common genes, *MAPK1*, *PRKCB*, *PRKCG*, *HDAC1*, *HTR1A*, *HTR2A*, *HTR2C*, and *HTR7*, shared across all three compounds and stroke. These genes are involved in inflammation, nerve signaling, and gene regulation, which are important processes in stroke. CHEMBL3613624 covered all eight genes, suggesting strong potential for further development. Overall, this integrated QSAR–docking–simulation–network approach offers a robust foundation for experimental validation and further development of cofilin-targeted therapeutics for stroke.

## Figures and Tables

**Table 1:** Performance of the best QSAR models for each descriptor set, showing training/test regression metrics and test classification metrics.

Descriptor Set	MACCS	CDK	PubChem	Substructure
Algorithm	Bagging	Ridge (Pace-like)	Gradient Boosting	Gradient Boosting
R (Train)	0.975	0.999	0.975	0.886
R^2^ (Train)	0.943	0.999	0.947	0.784
RMSE (Train)	0.333	0.032	0.32	0.647
MAE (Train)	0.236	0.015	0.23	0.453
R (Test)	0.883	0.835	0.834	0.826
R^2^ (Test)	0.764	0.674	0.661	0.662
RMSE (Test)	0.673	0.792	0.807	0.806
MAE (Test)	0.501	0.571	0.54	0.559
Precision	1	1	1	1
Recall	0.684	0.789	0.632	0.789
F1 Score	0.812	0.882	0.774	0.882

**Table 2:** Comparison of model performance before and after hyperparameter optimization across four descriptor sets (CDK, MACCS, PubChem, and substructure). Metrics include R^2^, RMSE, and MAE (all reported as mean ± SD). Post-optimization results also include the best-performing parameter configurations for each model.

Before Hyperparameter Tuning				
Descriptors	Model	RÂ^2^ Mean Â ± SD	RMSE Mean Â ± SD	MAE Mean Â ± SD
CDK	Random Forest	0.5674 Â ± 0.2124	0.8373 Â ± 0.2109	0.6437 Â ± 0.1338
CDK	SVR	0.5805 Â ± 0.2233	0.8239 Â ± 0.1954	0.6377 Â ± 0.1288
CDK	Gradient Boosting	0.4894 Â ± 0.3605	0.8795 Â ± 0.2641	0.6442 Â ± 0.1466
CDK	KNN	0.4489 Â ± 0.3967	0.9237 Â ± 0.2292	0.6935 Â ± 0.1483
CDK	PLS Regression	0.5272 Â ± 0.3817	0.8446 Â ± 0.2748	0.6219 Â ± 0.1766
MACCS	Random Forest	0.5414 Â ± 0.2264	0.8570 Â ± 0.2082	0.6491 Â ± 0.1374
MACCS	SVR	0.4871 Â ± 0.2934	0.8979 Â ± 0.2244	0.6902 Â ± 0.1638
MACCS	Gradient Boosting	0.3942 Â ± 0.2867	0.9875 Â ± 0.1955	0.7467 Â ± 0.1289
MACCS	KNN	0.3999 Â ± 0.4142	0.9489 Â ± 0.2511	0.7174 Â ± 0.1848
PubChem	Random Forest	0.5768 Â ± 0.2296	0.8206 Â ± 0.2157	0.6109 Â ± 0.1319
PubChem	SVR	0.5556 Â ± 0.2236	0.8486 Â ± 0.1984	0.6498 Â ± 0.1362
PubChem	Gradient Boosting	0.5281 Â ± 0.2857	0.8562 Â ± 0.2406	0.6218 Â ± 0.1451
Substructure	Random Forest	0.4756 Â ± 0.2937	0.9098 Â ± 0.2312	0.6941 Â ± 0.1582
Substructure	SVR	0.5276 Â ± 0.2665	0.8667 Â ± 0.2321	0.6845 Â ± 0.1626
Substructure	KNN	0.4212 Â ± 0.3797	0.9486 Â ± 0.2470	0.7390 Â ± 0.1915
Substructure	Gradient Boosting	0.4772 Â ± 0.3235	0.9068 Â ± 0.2419	0.6854 Â ± 0.1726
**After Hyperparameter Tuning**				
**Descriptors**	**Model**	**RÂ^2^ Mean Â ± SD**	**RMSE Mean Â ± SD**	**MAE Mean Â ± SD**
CDK	Random Forest	0.5642 Â ± 0.2298	0.8341 Â ± 0.2174	0.6447 Â ± 0.1427
CDK	SVR	0.5805 Â ± 0.2233	0.8239 Â ± 0.1954	0.6377 Â ± 0.1288
CDK	Gradient Boosting	0.5059 Â ± 0.3210	0.8725 Â ± 0.2622	0.6438 Â ± 0.1530
CDK	KNN	0.5150 Â ± 0.2978	0.8727 Â ± 0.1976	0.6659 Â ± 0.1156
CDK	PLS Regression	0.4772 Â ± 0.3149	0.9068 Â ± 0.2265	0.7244 Â ± 0.1625
MACCS	Random Forest	0.5575 Â ± 0.2120	0.8447 Â ± 0.2076	0.6596 Â ± 0.1384
MACCS	SVR	0.5042 Â ± 0.2505	0.8932 Â ± 0.1906	0.7230 Â ± 0.1437
MACCS	Gradient Boosting	0.4473 Â ± 0.2564	0.9431 Â ± 0.2093	0.7375 Â ± 0.1509
PubChem	Random Forest	0.5826 Â ± 0.2291	0.8107 Â ± 0.2113	0.6184 Â ± 0.1377
PubChem	SVR	0.5591 Â ± 0.1905	0.8533 Â ± 0.1897	0.6950 Â ± 0.1282
PubChem	Gradient Boosting	0.5587 Â ± 0.2424	0.8307 Â ± 0.2395	0.6277 Â ± 0.1501
Substructure	Random Forest	0.5029 Â ± 0.2550	0.8944 Â ± 0.2190	0.6969 Â ± 0.1513
Substructure	SVR	0.5285 Â ± 0.2487	0.8746 Â ± 0.2138	0.7081 Â ± 0.1515
Substructure	Gradient Boosting	0.4765 Â ± 0.3351	0.9045 Â ± 0.2388	0.7020 Â ± 0.1669

**Table 3:** Summary of key overlapping genes between top repurposed compounds and stroke-associated gene sets, including functional clusters, stroke-related roles, and significantly enriched pathways (FDR < 0.0001) from network pharmacology analysis.

Gene	Functional Cluster	Stroke-Related Role	Enriched Pathways (FDR < 0.0001)
*MAPK1*	Intracellular signaling kinase	Neuroinflammation, apoptosis, BBB integrity	MAPK signaling, inflammatory mediator regulation
*PRKCB*	Intracellular signaling kinase	Inflammatory cascades, neuronal death	Calcium signaling, *PKC* signaling
PRKCG	Intracellular signaling kinase	BBB regulation, oxidative stress	Calcium signaling, *PKC* signaling
*HDAC1*	Epigenetic regulator	Chromatin remodeling, neuronal survival	Histone modification, DNA repair
*HTR1A*	Serotonin receptor	Vasodilation, synaptic plasticity	Serotonergic synapse
*HTR2A*	Serotonin receptor	Vasodilation, platelet aggregation	Serotonergic synapse
*HTR2C*	Serotonin receptor	Cognitive recovery, mood modulation	Serotonergic synapse
*HTR7*	Serotonin receptor	Neurovascular regulation, post-stroke depression	Serotonergic synapse

## Data Availability

The original contributions presented in this study are included in the article/Supplementary Material. Further inquiries can be directed at the corresponding author(s).

## Supplementary Materials

The following supporting information can be downloaded at: https://www.mdpi.com/article/10.3390/ph18091323/s1, Tables S1–S42: Literature collection and statistics result (provided in Excel format); Figures S1–S8: Results analysis shown in images from SHAP analysis, chemical structures, ligand properties from molecular dynamics study, and gene ontology image.
