# Supplementary material for: Repurposing Cofilin-Targeting Compounds for Ischemic Stroke Through Cheminformatics and Network Pharmacology
Source: Pharmaceuticals (Basel). 2025 Sep 4;18(9):1323. doi: 10.3390/ph18091323 (PMC12472226; doi:10.3390/ph18091323)
Supplement: Supplementary file 1 [file pharmaceuticals-18-01323-s001.zip › pharmaceuticals-3808310-supplementary Figures.pdf]

Supplementary Figures

Repurposing Cofilin-Targeting Compounds for Ischemic Stroke through Cheminformatics and Network Pharmacology

Saleh I. Alaqel [1,2,*], Abida Khan [1,3], Mashael N. Alanazi [1], Naira Nayeem [1], Hayet Ben Khaled [1] and Mohd Imran [1,3]

1. Department of Pharmaceutical Chemistry, College of Pharmacy, Northern Border University, Rafha 91911, Saudi Arabia. (saleh.alaqel@nbu.edu.sa, S.I.A.; abeda.mohammed@nbu.edu.sa, A.K.; Mashael.Alanazi@nbu.edu.sa, M.N.A.; nurah.akml@nbu.edu.sa, N.N.; H.B.K.,, hayat.khaled@nbu.edu.sa; M.I., mohammad.baks@nbu.edu.sa)
2. King Salman Center for Disability Research, Riyadh 11614, Saudi Arabia.
3. Center For Health Research, Northern Border University, Arar, Saudi Arabia.

*Correspondence: saleh.alaqel@nbu.edu.sa

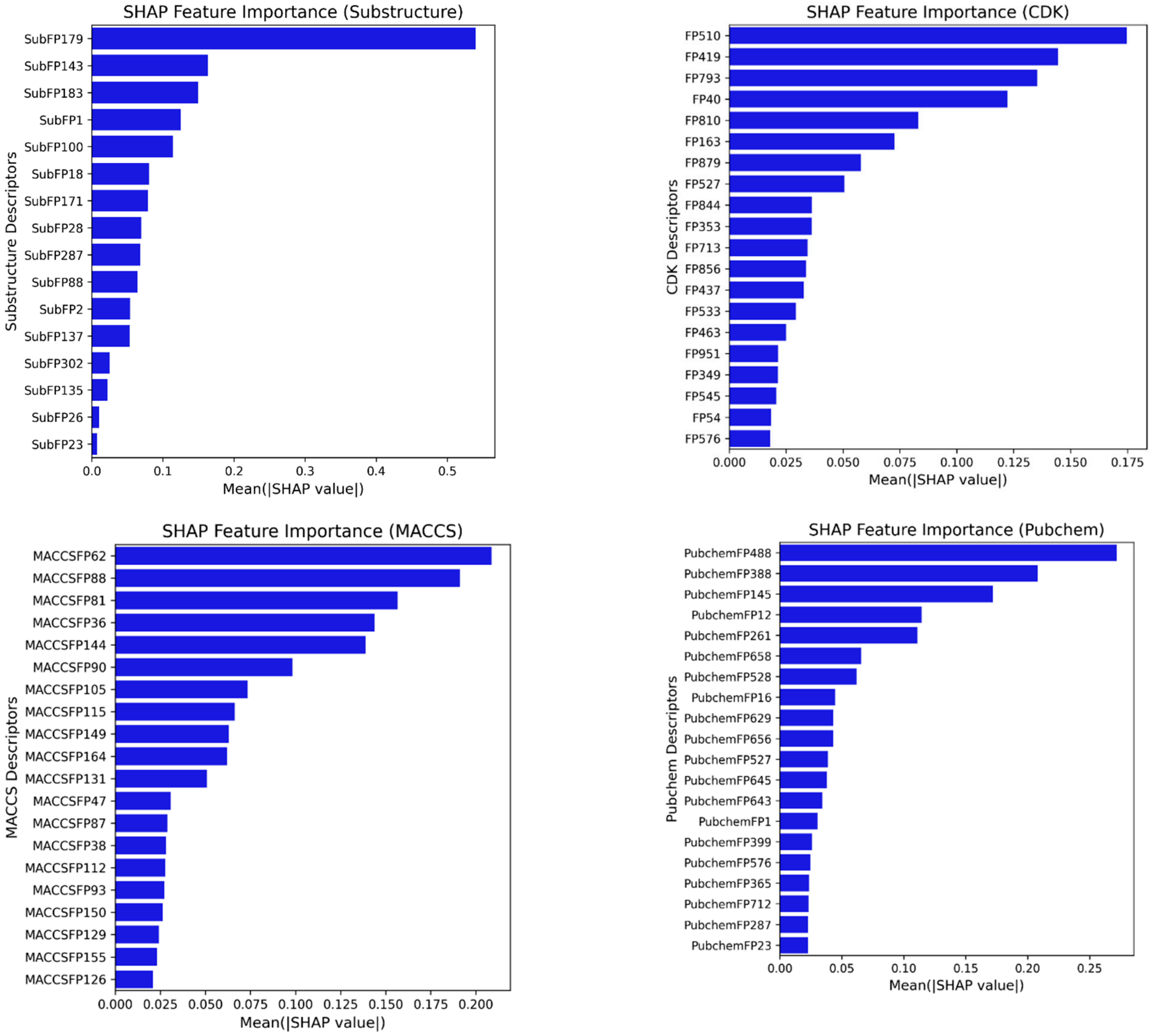

Figure S1: SHAP features importance description of all selective descriptors.

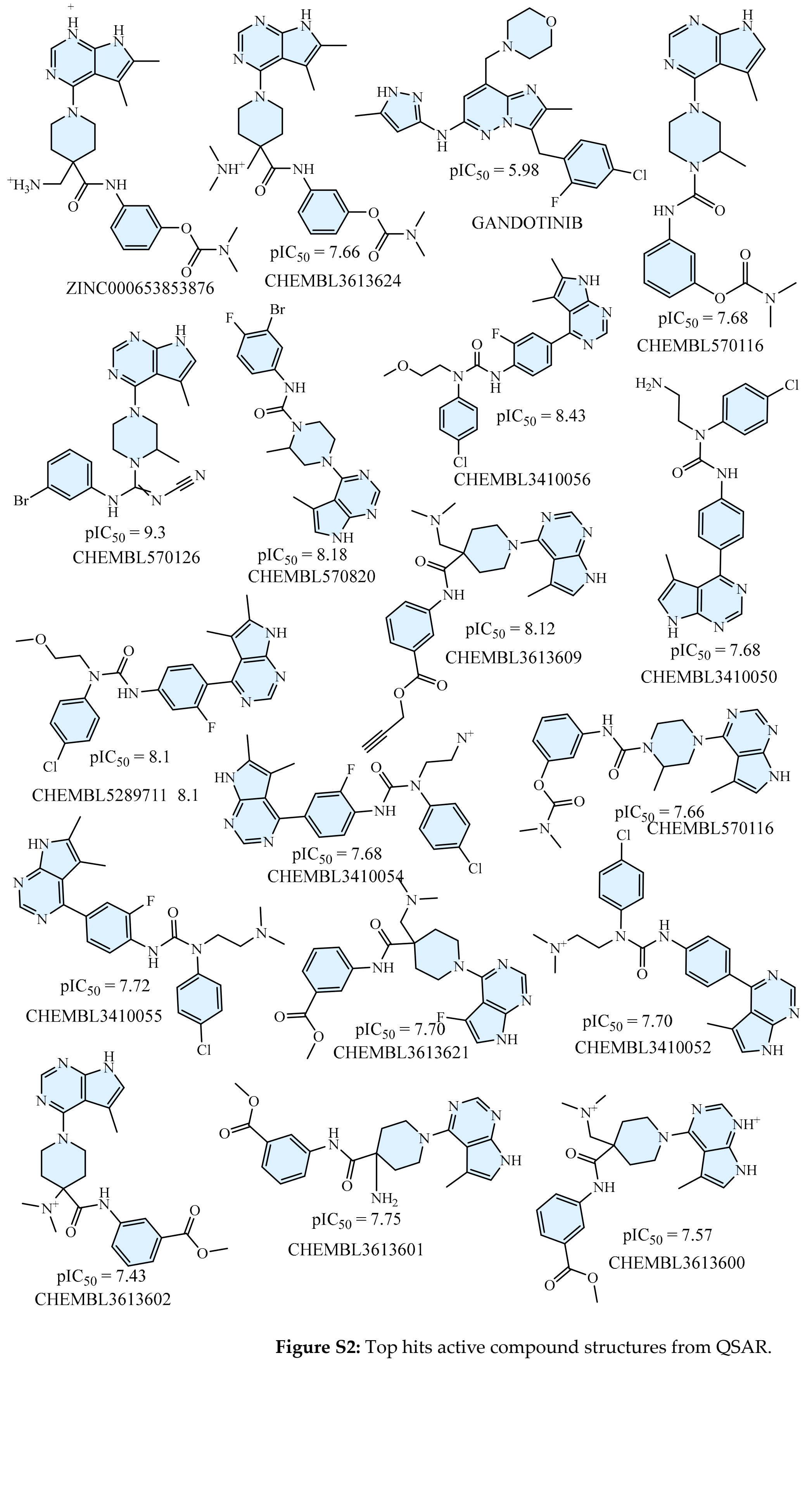

Figure S2: Top hits active compound structures from QSAR.

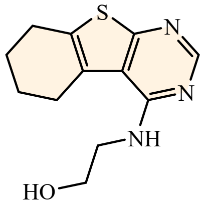

pIC_{50} = 4

CHEMBL1269735

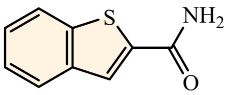

pIC_{50} = 4

CHEMBL538197

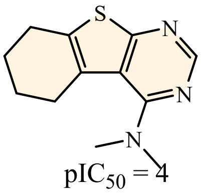

pIC_{50} = 4

CHEMBL1165129

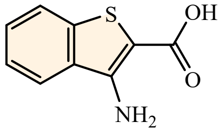

pIC_{50} = 4

CHEMBL3219017

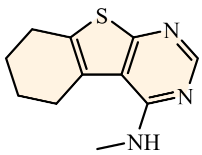

pIC_{50} = 4

CHEMBL1836481

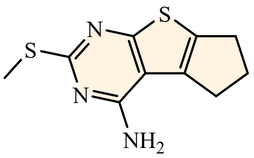

pIC_{50} = 4

CHEMBL1836476

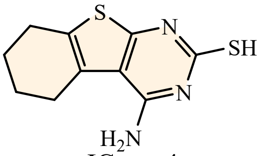

pIC_{50} = 4

CHEMBL1836477

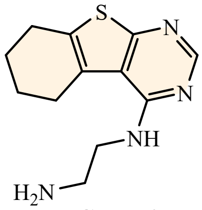

pIC_{50} = 4

CHEMBL1836482

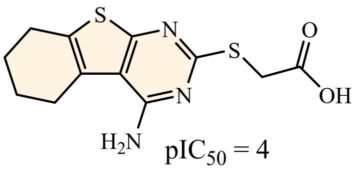

pIC_{50} = 4

CHEMBL1836478

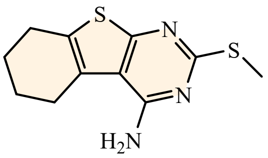

pIC_{50} = 4

CHEMBL575039

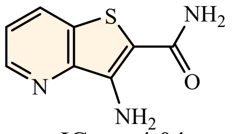

pIC_{50} = 4.04

CHEMBL3219021

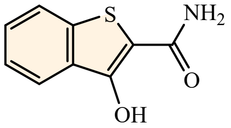

pIC_{50} = 4

CHEMBL3219020

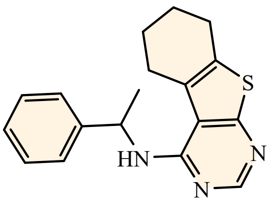

pIC_{50} = 4

CHEMBL1836483

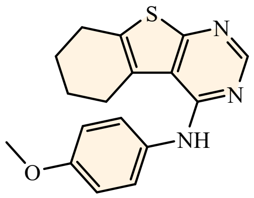

pIC_{50} = 4

CHEMBL1165229

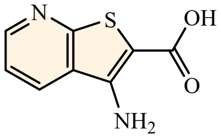

pIC_{50} = 4

CHEMBL3219022

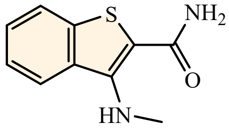

pIC_{50} = 4.47

CHEMBL3219018

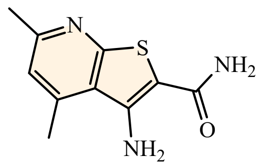

pIC_{50} = 5.05

CHEMBL377085

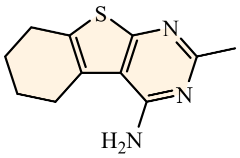

pIC_{50} = 4

CHEMBL1836474

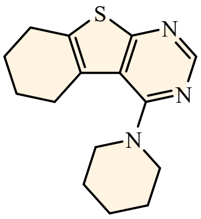

pIC_{50} = 4

CHEMBL1836484

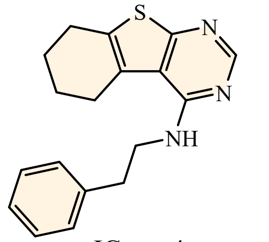

pIC_{50} = 4

CHEMBL1271896

Figure S3: Top hits inactive compound structures from QSAR.

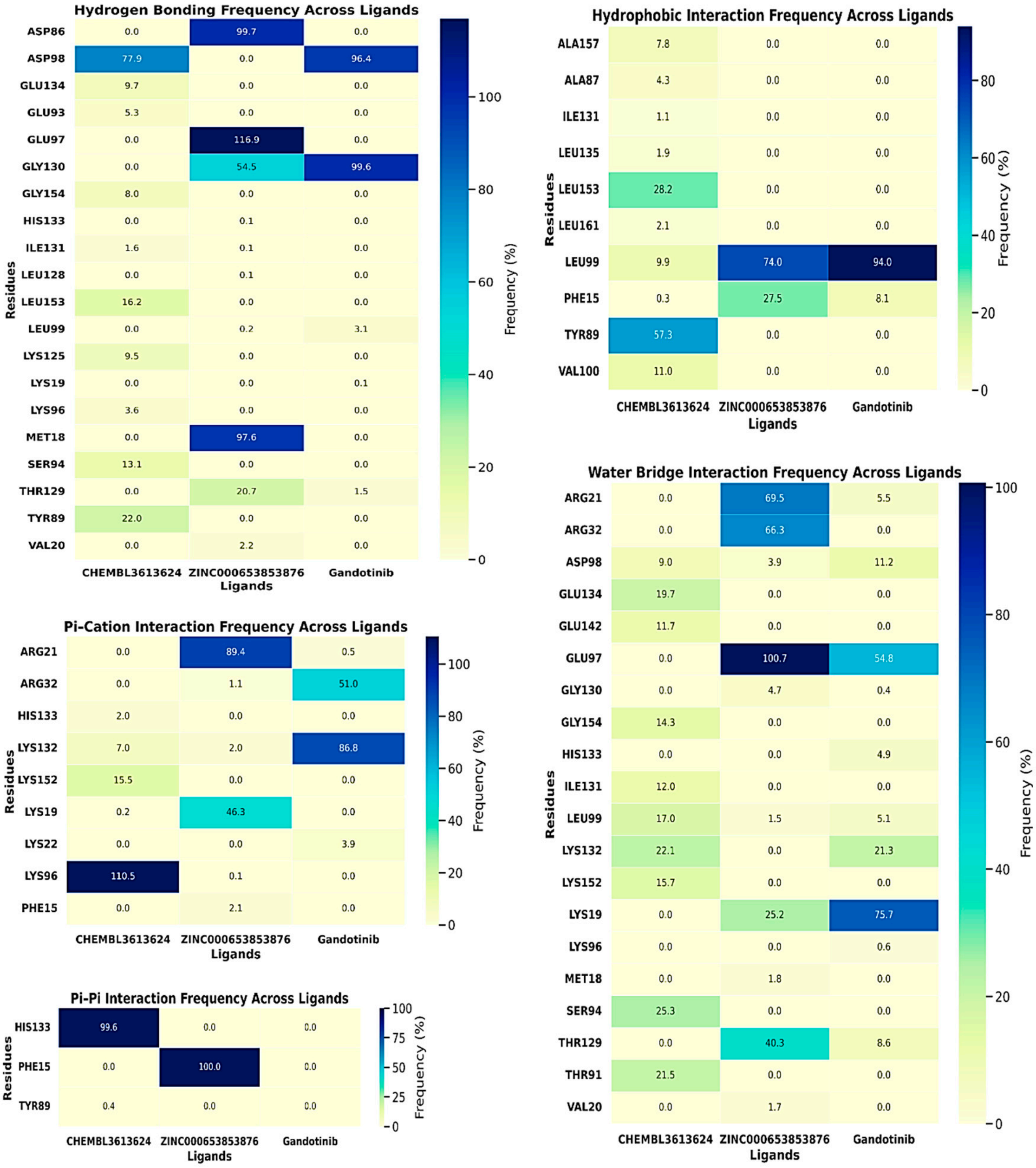

Figure S4. Interaction frequency maps of cofilin with CHEMBL3613624, ZINC000653853876, and Gandotinib during molecular dynamics simulation. Heatmaps show hydrogen bonding, hydrophobic interactions, water bridges, π-cation, and π–π stacking interactions across key residues.

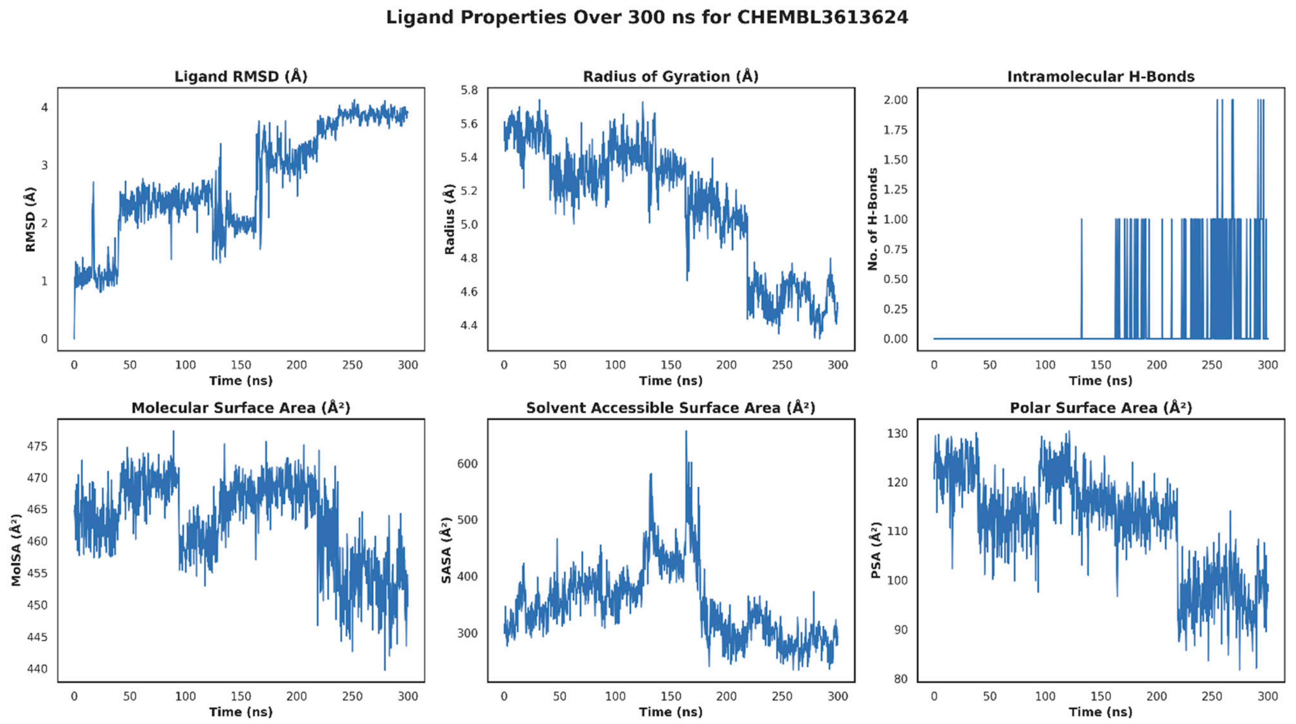

Figure S5: Ligand properties analysis over 300ns for compound CHEMBL3613624.

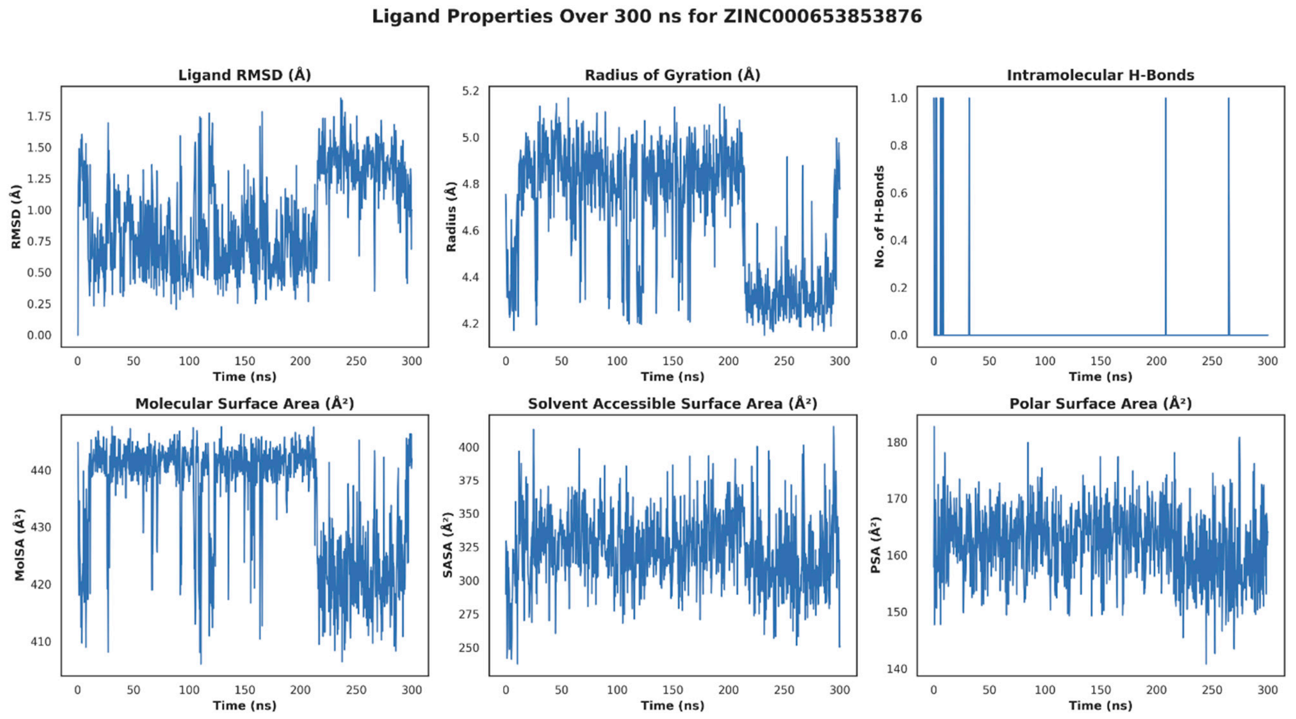

Figure S6: Ligand properties analysis over 300ns for compound ZINC000653853876.

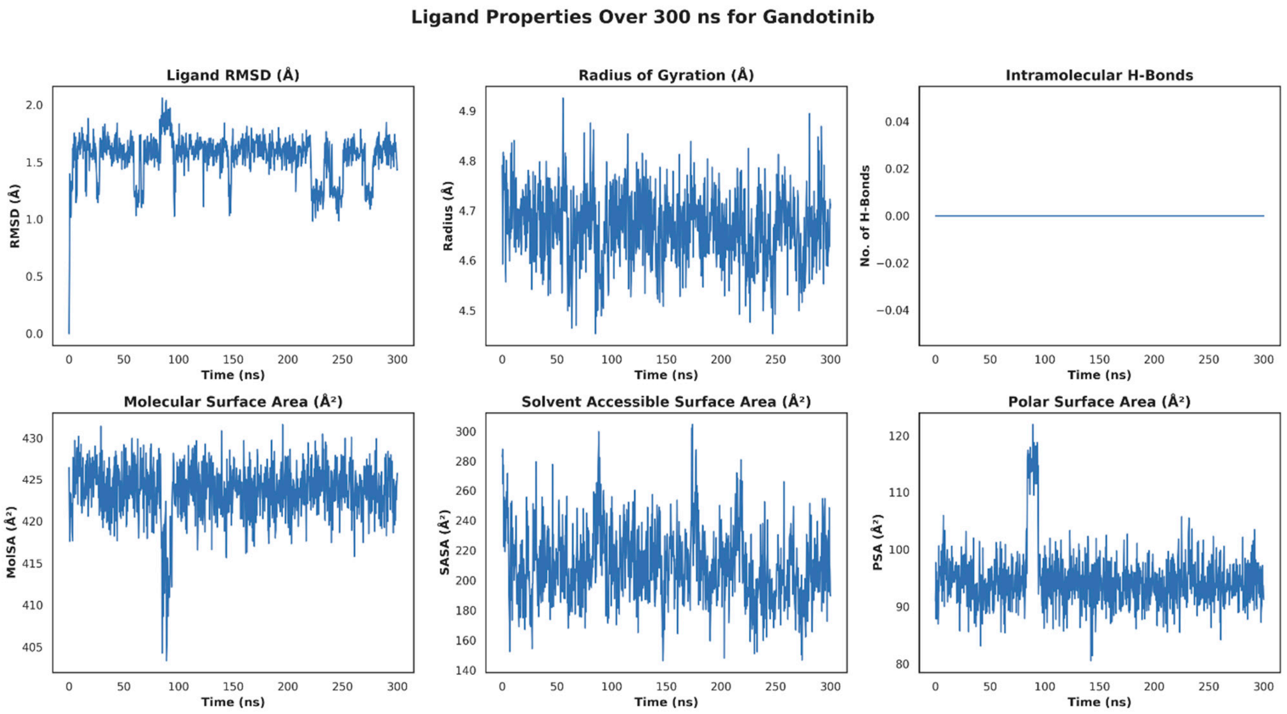

Figure S7: Ligand properties analysis over 300ns for compound Gandotinib.

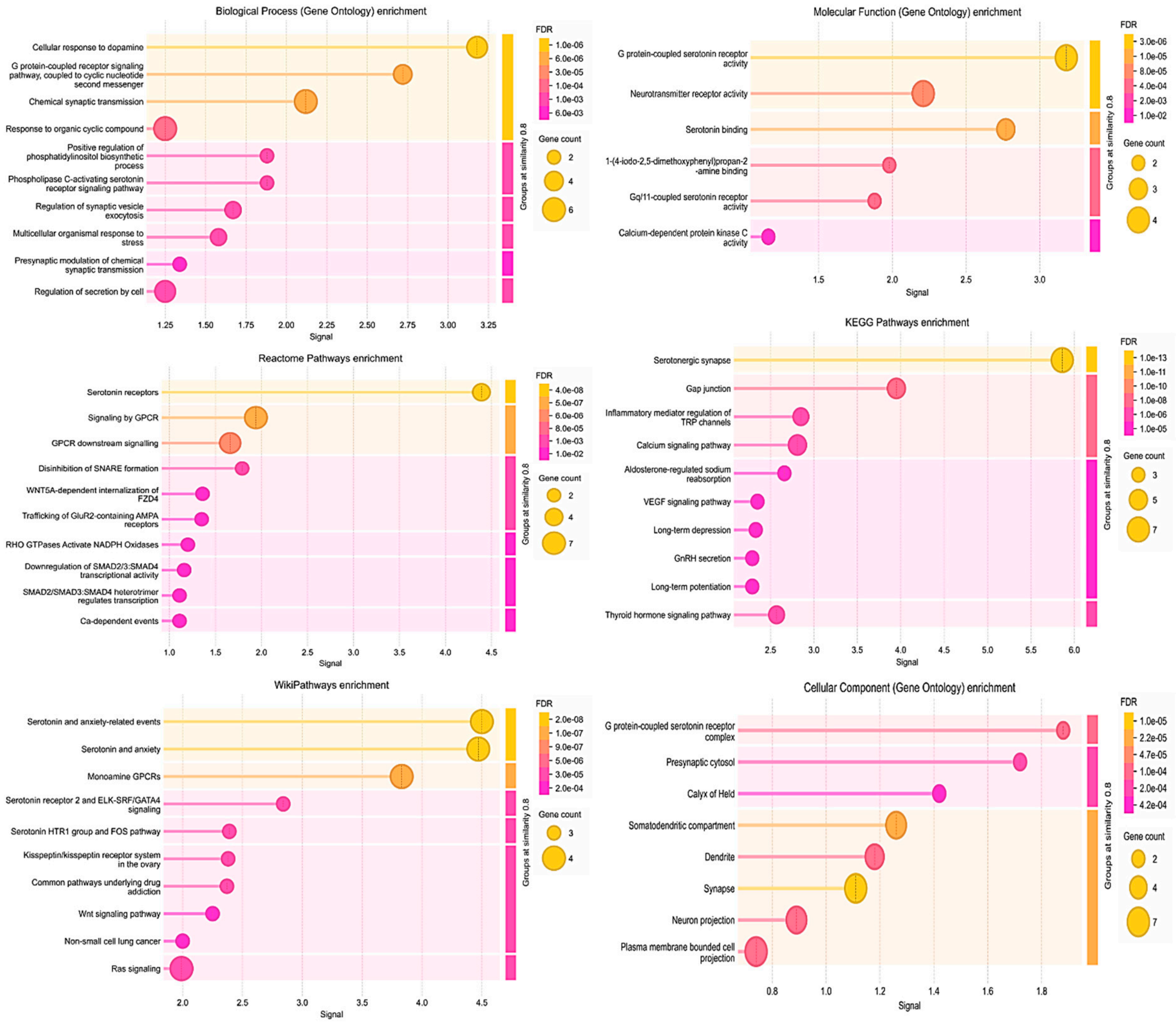

Figure S8. Gene ontology analysis of different molecular and functional annotations.
